# Understanding Public Perceptions of Lung Cancer in China: Infodemiology Study of Baidu Index and Weibo Posts

**DOI:** 10.2196/85058

**Published:** 2026-05-05

**Authors:** Zongyuan Li, Jian Zhang, Yiming Fan, Nan Chen, Lunxu Liu

**Affiliations:** 1 Department of Thoracic Surgery and Institute of Thoracic Oncology, West China Hospital of Sichuan University, No. 37, Guoxue Alley, Wuhou District, Chengdu, 610041, China, +86 28 85422494

**Keywords:** lung neoplasms, Baidu index, Weibo, infodemiology, public health, topic modeling, spatial analysis, social media, sentiment analysis

## Abstract

**Background:**

In China, lung cancer remains a major public health concern and accounts for a substantial proportion of cancer-related deaths nationwide. However, limited research has examined public perceptions of lung cancer in the digital sphere, where health-related information is increasingly disseminated and accessed.

**Objective:**

This study aims to systematically examine patterns of public attention and perceptions toward lung cancer in China by integrating search engine query data and social media content, thereby enhancing current understanding of web-based health information dynamics related to lung cancer.

**Methods:**

Data were collected from Baidu Index (BI) (2011‐2025) and Sina Weibo (2010‐2025) to represent web-based search behavior and social media discourse on lung cancer, respectively. Spatiotemporal patterns of BI, per capita Baidu Index (PBI), and Weibo posts were examined to capture temporal trends and spatial variations. Additionally, the spatial autocorrelation of PBI was assessed using global and local Moran *I* statistics. PBI-related explanatory variables were assessed using a spatial panel Durbin model. Topic modeling and lexicon-based sentiment analysis were applied to Weibo content to uncover thematic evolution and emotional polarity across years, sex/organization groups, and user types.

**Results:**

Public attention toward lung cancer, as reflected by BI, increased initially, peaked in 2019, and subsequently declined, whereas Weibo discussions demonstrated a fluctuating but generally upward trend before stabilizing after 2022. Similar temporal patterns were observed across most provinces. Significant spatial heterogeneity was identified, with higher BI levels concentrated in eastern coastal regions and persistently lower levels in western and southwestern provinces. Spatial autocorrelation analysis revealed stable positive clustering over time, with low-low clusters particularly concentrated in southwestern regions such as Guangxi, and no significant high-high clusters were detected. Panel spatial regression analyses indicated that the provincial PBI was positively associated with gross domestic product (GDP) per capita and average years of education per capita, but negatively associated with the urbanization rate. Moreover, significant spatial spillover effects were observed, suggesting that socioeconomic factors were associated not only with local public attention but also with that of neighboring regions. Topic modeling revealed a clear thematic evolution over time. Although personal experiences initially dominated web-based discourse, discussions progressively shifted toward health care service–related issues, which became the most prominent theme by 2025. Sentiment analysis indicated an overall positive emotional tone throughout the study period, with “Good” and “Disgust” representing the predominant positive and negative emotions, respectively. Emotional expression varied across demographic groups and user types, with noticeable differences in both intensity and temporal trends.

**Conclusions:**

This study offers a comprehensive overview of public attention and discourse on lung cancer in China’s digital landscape, providing valuable evidence to inform targeted health communication and policy interventions.

## Introduction

Lung cancer remained the most frequently diagnosed malignancy and the leading cause of cancer-related mortality in 2022, accounting for approximately 2.5 million new cases worldwide [1,2]. Despite a global decline in age-standardized lung cancer incidence and mortality rates [3], the burden of lung cancer in China remains high and continues to rise [4]. In 2021, China accounted for approximately 40% of global lung cancer deaths, with over 800,000 fatalities [3,5]. China also bears the highest share of the global cancer economic burden, with lung cancer representing the costliest type [6]. This growing disease burden presents a substantial public health challenge, particularly given China’s aging population and high prevalence of tobacco use [7]. According to Surveillance, Epidemiology, and End Results 21 data (2015‐2021), the 5-year relative survival rate for lung cancer is 28.1%, with more than half of cases diagnosed at a distant stage and only about one-quarter at a localized stage [8]. Randomized controlled trials have demonstrated that low-dose computed tomography screening significantly reduces lung cancer mortality [9,10]. Indeed, early diagnosis and appropriate treatment are critical for improving lung cancer prognosis. However, increasing evidence suggests that inadequate public awareness and widespread misperceptions about lung cancer risk factors, symptoms, screening, and treatment options contribute to delays in diagnosis and underutilization of health care services [11,12]. A recent cross-sectional survey of 31,184 urban Chinese residents revealed that 76.12% of respondents acknowledged having limited knowledge of lung cancer [13]. Moreover, pervasive stigma and misinformation surrounding lung cancer may further deter individuals from engaging in health-seeking behavior and receiving timely treatment [14,15].

Therefore, enhancing public awareness and knowledge of lung cancer has emerged as a key priority in global cancer-control strategies. To this end, understanding the current attention and prevailing attitudes toward lung cancer is a prerequisite for developing effective public awareness strategies that address existing knowledge gaps and prioritize areas for intervention. Relatively few studies have examined these issues in the Chinese population. For instance, an analysis of 481 questionnaires completed by the offspring of lung cancer patients indicated insufficient knowledge, favorable attitudes, and inadequate practice regarding lung cancer risk [16]. A nationwide survey of 2093 health care practitioners across 706 hospitals in China revealed substantial disparities in knowledge, attitudes, and practices concerning lung cancer palliative care, highlighting the pressing need for comprehensive training initiatives [17]. Nonetheless, prior research in the Chinese population has predominantly relied on regionally confined samples, narrowly defined groups (eg, the offspring of lung cancer patients and health care practitioners), and structured questionnaires, often using small sample sizes and limited timeframes, thereby restricting the generalizability of their findings [13,16-18]. Consequently, there remains a notable lack of nationally representative studies that comprehensively investigate both macro-level public attention and micro-level discursive practices related to lung cancer across public domains in China.

With the widespread adoption of digital platforms in China, an increasing number of individuals are using search engines and social media to access and share health-related information [19]. According to the 56th Statistical Report on China’s Internet Development, as of June 2025, China had 1.123 billion internet users and an internet penetration rate of 79.7%, with social media users reaching 1.107 billion (representing 98.6% of the web population) [20]. In China’s search engine market, Baidu holds a dominant position with a penetration rate of 90.9%, serving as the primary platform through which users access web-based information [21]. The Baidu Index (BI), derived from massive user behavior data, quantifies search volumes for specific keywords over time and geographic regions, generating a relative index value [22]. BI reflects users’ active search interests and has been widely used in public health research as a proxy for monitoring information-seeking behavior and public awareness [23]. Regarding social media, Weibo, launched by Sina Corporation in 2009, has become China’s leading microblogging platform, with 587 million monthly and 257 million daily active users as of September 2024, and serves as a major venue for real-time content sharing and public discourse [24]. Prior studies have leveraged these web-based data to monitor health behavior trends and identify information gaps across diseases, offering real-time insights into population-level perceptions [19,22,23,25,26]. However, few studies have systematically analyzed how lung cancer is represented and discussed via web in China. To address this gap, this study aims to investigate public perceptions of lung cancer in China using a framework that integrates BI data from large-scale search queries and posts from the social media platform Weibo. This dual-perspective approach combines information-seeking behavior with social communication dynamics, providing a more comprehensive and multidimensional understanding of public attention than single-source or traditional surveillance methods. By examining public attention and web-based discourse, this study seeks to provide evidence for health communication strategies and public health initiatives on lung cancer in China, with the potential to enhance awareness, promote earlier detection, and strengthen disease management.

## Methods

**Figure 1.**
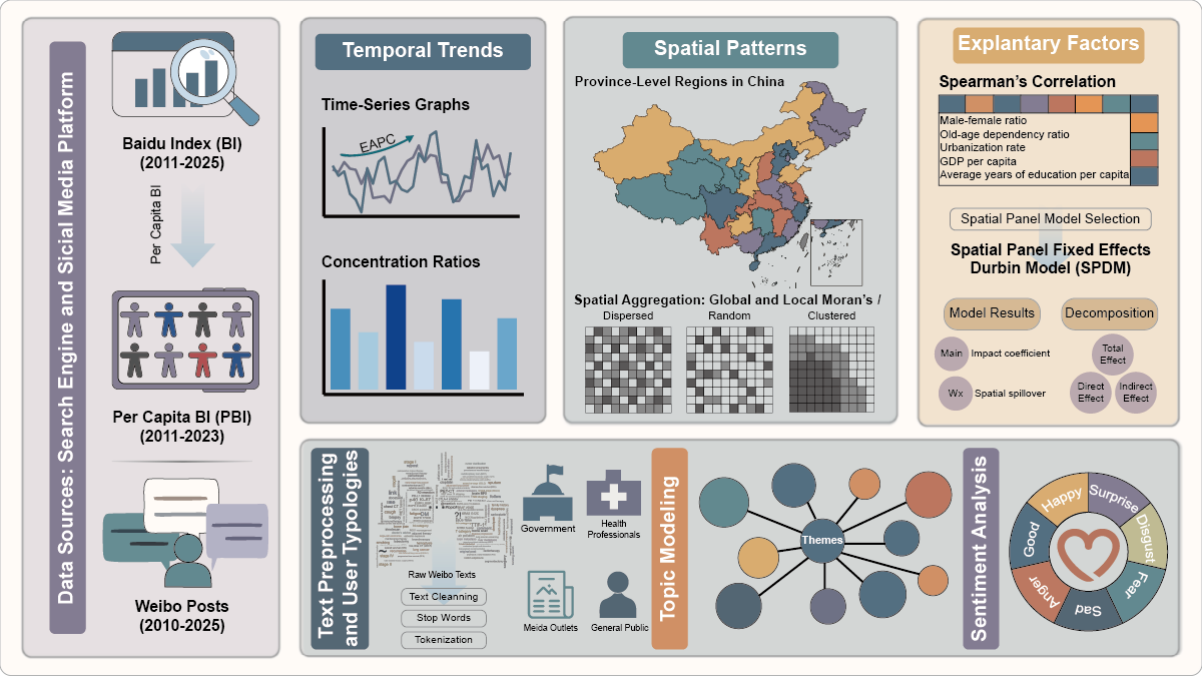
Overview of study design and analytical workflow. BI: Baidu Index; EAPC: estimated annual percent change; GDP: gross domestic product; PBI: per capita Baidu Index.

An overview of the study design and analytical workflow is presented in Figure 1.

### Data Sources

Search engine data related to lung cancer was obtained from the official website of BI. Researchers can use this platform to quantify the frequency of keyword searches on Baidu across different regions and over defined temporal spans. In this study, daily BI values for Chinese-language keywords referring to lung cancer, including “lung cancer,” “neoplasms of the lung,” “lung adenocarcinoma,” and “lung squamous cell carcinoma” were collected for 31 provincial-level regions (22 provinces, 5 autonomous regions, and 4 municipalities) in mainland China between January 1, 2011, and August 31, 2025. Hong Kong, Macao, and Taiwan were excluded from the analysis. Monthly and yearly average BI values were defined as the mean of daily BI values within each respective month or year. To estimate search activity per capita within each province and enable regional comparisons, the per capita Baidu Index (PBI) was calculated as BI divided by the year-end population, scaled per 10,000 individuals [26].

We retrieved relevant original posts (also referred to as thread posts) from Weibo using its keyword-based advanced search function. Reposts (shares) and comment replies were not included in the dataset. Combinations of Chinese keywords related to lung cancer, including “lung cancer,” “neoplasms of the lung,” “lung adenocarcinoma,” and “lung squamous cell carcinoma,” were selected to retrieve relevant posts between January 1, 2010 (roughly 4 months after Weibo’s launch in China), and August 31, 2025. These search keywords were primarily determined based on the researchers’ prior knowledge. In addition, user-related metadata (eg, username, sex/organization, personal description, avatar, verification status, number of posts, follower number, following number, and other information) were also collected. In this study, Weibo users were categorized as male (35,792/105,549, 33.91%), female (53,800/105,549, 50.97%), or organization (15,957/105,549, 15.12%) according to their self-reported sex and verification information provided in their profiles (Table 1) [27]. Given that all accounts report sex and organizational accounts can be separately identified via verification information, we classified them as a distinct category. Institutional accounts were not treated as a sex category but rather as a distinct user type to better capture user heterogeneity. Based on user information verified by Weibo, users were also categorized into 4 types, including government (4478/105,549, 4.24%), health professionals (4093/105,549, 3.88%), media outlets (3827/105,549, 3.63%), and the general public (93,151/105,549, 88.25%) (Table 1). We performed text preprocessing to clean the dataset as a preparatory step to text-mining analysis (Multimedia Appendix 1 [26-33] provides further details). Following duplicate removal and text preprocessing, a total of 268,756 original posts from 105,549 unique Weibo users were retained for subsequent analysis.

**Table 1.** Classification of Weibo users in this study by sex/organization and user type. Classification of Weibo users was based on verification information and self-reported profile details. Examples are illustrative and anonymized; institutional names are shown only as representative categories, and no personal identifiers are disclosed.

Group	Description	Examples	Users (n=105,549), n (%)	Posts (n=268,756), n (%)
Sex/organization				
Male	Self-reported male users	Mr. Zhang (verified personal account, health blogger, male)	35,792 (33.91)	94,959 (35.33)
Female	Self-reported female users	Ms. Li (verified personal account, writer, female)	53,800 (50.97)	80,291 (29.88)
Organization	Institutional users identified from verification information	Beijing Cancer Hospital official account	15,957 (15.12)	93,506 (34.79)
User type				
Government	Chinese governmental bodies at different levels	National Health Commission, Taoyuan County People’s Government (Hunan Province) official account	4478 (4.24)	25,818 (9.61)
Health professionals	Users with medical or scientific expertise, including clinicians, researchers, and health care providers	Oncologist at a tertiary hospital, TCM^a^ practitioner	4093 (3.88)	46,265 (17.21)
Media outlets	News organizations and journalists, including newspapers, broadcasters, and digital media	China Media Group, Renwu magazine	3827 (3.63)	33,480 (12.46)
General public	Users outside the above categories, typically unverified individuals sharing personal views or experience	Ordinary Weibo user, patient sharing experience	93,151 (88.25)	1,63,193 (60.72)

a TCM: traditional Chinese medicine.

Provincial-level demographic and socioeconomic explanatory variables were sourced from the China Statistical Yearbook (2011‐2023) and corresponding provincial statistical yearbooks. The demographic variables comprised the male-female ratio, old-age dependency ratio (the proportion of individuals aged 65 years and older to those aged 15‐64 years), and urbanization rate (the share of the urban population in the total population at year-end). Meanwhile, the socioeconomic factors considered were gross domestic product (GDP) per capita, an indicator of economic development, and average years of education per capita, which reflect the educational level of the region (Multimedia Appendix 1 [26-33] provides details on calculation). Due to the publication lag of official statistics, complete and consistent provincial-level demographic and socioeconomic data were available only through 2023, with data for 2024 onward unavailable at the time of analysis. The descriptive statistical summaries of these variables can be found in Figure S1 and Table S1 in Multimedia Appendix 1 [26-33].

### Statistical Analysis

An integrated framework was developed to investigate macro-level public attention and micro-level web-based discourse on lung cancer, comprising four analytical dimensions that addressed temporal trends, spatial patterns, a spatial panel econometric model, and text-mining analysis. All analyses were conducted with R (version 4.4.2; R Foundation for Statistical Computing, Vienna, Austria). A two-tailed *P* value less than .05 was regarded as statistically significant.

First, temporal trends in BI, PBI, and the number of Weibo posts were depicted using time-series graphs, and provincial BI and PBI from 2011 to 2023 were subsequently assessed through estimated annual percent change (EAPC). In the time-series graphs, in addition to displaying mean values with point-line plots, locally weighted regression smoothing and generalized linear models were fitted using the base R “stats” package (version 4.5.1) to further characterize temporal trends. Details of the EAPC calculation are provided in Multimedia Appendix 1 [26-33]. To assess temporal aggregation of the BI for lung cancer, we applied the concentration ratio method (detailed in Multimedia Appendix 1) [26-33]. The index M ranges from 0 to 1; larger values denote stronger aggregation, while values below 0.3 indicate a relatively uniform distribution over time. This metric captures within-year seasonality rather than multi-year or long-term structural trends.

Second, we examined the spatial distribution patterns and spatial autocorrelation of search engine activity related to lung cancer. In this study, we defined the spatial weight matrix using the widely used Rook adjacency method, under which 2 provinces with a common boundary were assigned a weight of 1 and those without a boundary a weight of 0 [34]. Hainan Province, being an island without direct borders with other provinces, was designated as adjacent to Guangdong Province, the geographically closest province [26]. The spatial adjacency relationships of each province-level region in China are presented in Table S2 in Multimedia Appendix 1 [26-33]. Spatial autocorrelation analysis was used to assess the spatial clustering of the PBI for lung cancer [35]. Specifically, the global Moran *I* was applied to evaluate spatial dependence in provincial lung cancer PBI, while the local Moran *I* identified spatial aggregation or outlier patterns among adjacent provinces.

To examine the determinants of web-based search volume, this study applied Spearman rank correlation for initial association analysis and performed a variance inflation factor (VIF) test to detect potential multicollinearity. A threshold of VIF<10 was adopted as the criterion for variable retention, and all variables satisfied this criterion (Table S3 in Multimedia Appendix 1 [26-33]). Subsequently, following a series of model selection procedures (Figure S2 and Table S4 in Multimedia Appendix 1 [26-33]), the spatial panel Durbin model (SPDM) was used to quantify the effects, which explicitly incorporates spatial interactions and accounts for regional heterogeneity, compared with traditional panel models [28]. The SPDM model was estimated using data from 2011 to 2023 only, ensuring full temporal alignment between the dependent variables and all explanatory variables. A more detailed description of the spatial panel econometric model is provided in Multimedia Appendix 1 [26-33].

Moreover, we applied latent Dirichlet allocation (LDA) for topic modeling to identify lung cancer-related themes. LDA is a semiautomated and unsupervised machine learning method for computer-assisted content analysis that identifies latent semantic patterns in large text corpora [36,37]. An optimal number of topics was identified based on the LDA modeling results (Figure S3 in Multimedia Appendix 1 [26-33]), with *k* set to 50 to achieve a balance between interpretability and fine-grained thematic resolution. To interpret and label the LDA-generated topics and extract overarching themes, we examined 3 types of information for each topic, including top words, words both prevalent and distinctive, and representative Weibo posts. Topics were independently labeled by 2 coauthors using an open coding approach, followed by iterative comparison and discussion to reconcile differences and refine interpretations. Final themes were established through consensus. Furthermore, sentiment analysis was conducted to quantify the positive or negative sentiment expressed in lung cancer-related posts. Considering the need for transparent scoring and stable outputs in large-scale public health analysis, we used a lexicon-based approach instead of more complex deep learning or large language model-based methods. Sentiment was assessed using the DLUT-Emotion Ontology, a Chinese emotion lexicon developed by the Information Retrieval Laboratory at Dalian University of Technology [29,38]. Building upon internationally recognized emotion classification frameworks and integrating the linguistic characteristics of Chinese texts as well as existing emotion lexicons, this study categorizes emotions into 7 primary types, including “Happy,” “Good,” “Anger,” “Sad,” “Fear,” “Disgust,” and “Surprise.” Emotion Taxonomy and Example Lexemes of DLUT-Emotion Ontology are presented in Table S5 in Multimedia Appendix 1 [26-33]. After text preprocessing, tokens in each post were matched against the ontology. Each matched lexeme is annotated with an emotion category, polarity, and an intensity value ranging from 0 to 9. Sentiment scores were calculated by aggregating the intensity values of all matched tokens, weighted by their polarity (positive values for positive polarity and negative values for negative polarity). When multiple emotion categories were present within a single post, their contributions were cumulatively incorporated into the overall score. Continuous sentiment scores ranging from negative to positive were computed for each post, both for each emotion type and for an overall score. These scores have no predefined upper or lower bounds, as their magnitude depends on the cumulative number of matched tokens, their assigned intensity, and polarity. Sentiment scores were further analyzed by sex/organization, user category, and post theme.

### Ethical Considerations

This study analyzed publicly available, aggregated BI data and publicly accessible Weibo posts without direct interaction with individuals. Consistent with established practices in infodemiology research [22,27,39], studies analyzing anonymized, publicly available web data are generally considered exempt from formal ethics review; therefore, Institutional Review Board approval was not required. No identifiable personal information was collected or reported, and all data were analyzed in aggregated or deidentified form to ensure privacy and confidentiality. Because no human participants were recruited, informed consent and compensation were not applicable. No images or materials in the manuscript allow identification of individual users.

## Results

### Temporal Characteristics

**Figure 2.**
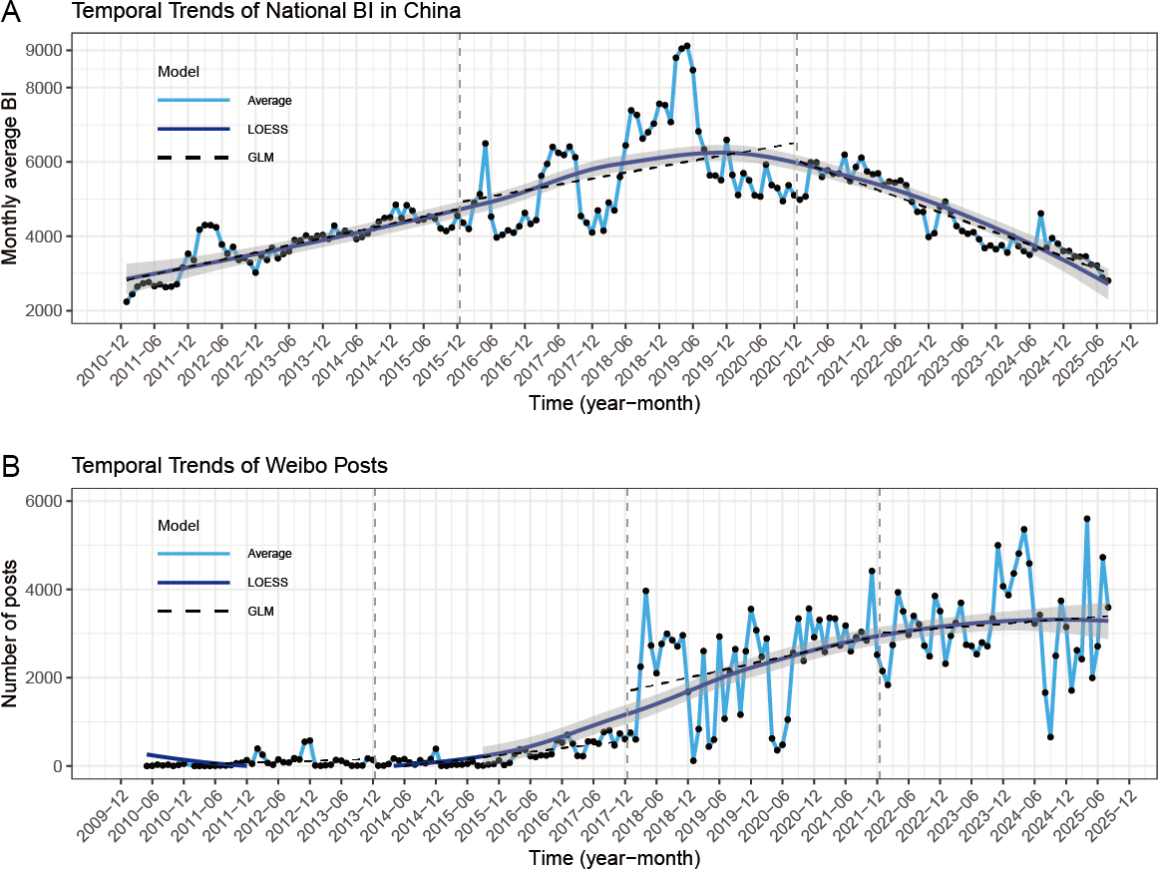
Temporal trends of national BI (A) and Weibo posts (B) for lung cancer in China. Monthly average values were reported, and LOESS, along with segmented GLM models, were applied to capture more detailed trends. BI: Baidu Index; GLM: generalized linear model; LOESS: locally weighted regression.

Figure 2A and B and Figure S4 in Multimedia Appendix 1 [26-33] present the temporal distribution of public attention to lung cancer, including the BI from January 2011 to August 2025 and the number of Weibo posts from January 2010 to August 2025. Over the course of approximately 15 years, the average BI was 4670.62. The temporal pattern of BI for lung cancer was characterized by an upward trend followed by a subsequent decline. The highest recorded BI was observed in May 2019, at 9,119.36. By August 2025, the BI had decreased to 2807.42. Besides, a comparable pattern of BI fluctuations was observed across the various province-level regions. Throughout the study period, the concentration ratios of BI ranged from 0.01 to 0.12, with the maximum value (*M*=0.12) recorded in 2019 (Table S6 in Multimedia Appendix 1 [26-33]). Moreover, the number of Weibo posts exhibited a fluctuating but overall upward trend starting in 2015, with growth gradually leveling off after 2022.

### Spatial Patterns

**Figure 3.**
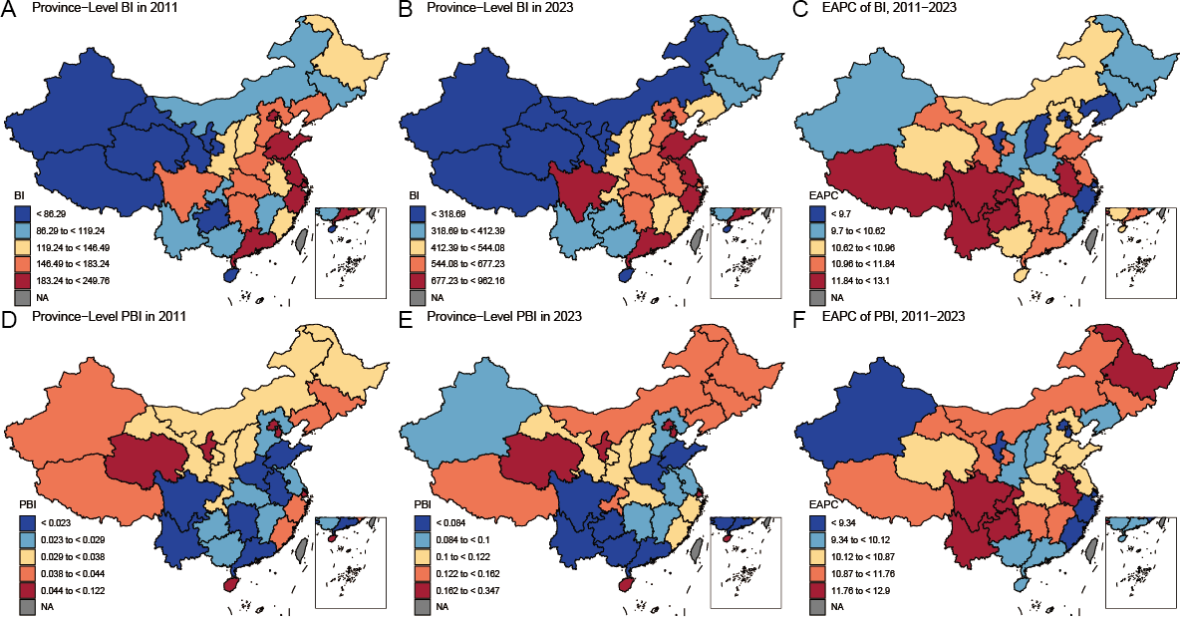
Spatial patterns of BI (A, B) and PBI (D, E) for lung cancer in 2011 and 2023, and EAPC (C, F) from 2011 to 2023, among provincial-level regions in China. BI: Baidu Index; EAPC: estimated annual percent change; PBI: per capita Baidu Index.

Figure 3A-F presents the spatial distribution of lung cancer BI and PBI across Chinese provinces from 2011 to 2023. Provinces with higher BI values for lung cancer include Beijing, Guangdong, Jiangsu, Shandong, and Zhejiang, which are primarily situated along China’s eastern and southeastern coast. Western and southwestern provinces, including Gansu, Ningxia, Qinghai, Tibet, Xinjiang, and Hainan, consistently exhibited the lowest BI levels. In contrast to BI, the PBI demonstrates a partially distinct spatial pattern, with elevated levels observed in Beijing, Hainan, Ningxia, Qinghai, Shanghai, and Tianjin. From 2011 to 2023, both the BI and PBI of lung cancer showed a general upward trend across Chinese provinces, with Sichuan and Yunnan showing the most pronounced increases in BI and PBI, respectively, as indicated by the highest EAPC values. Table 2 and Figure S5 in Multimedia Appendix 1 [26-33] present the global and local Moran *I* statistics for the PBI of lung cancer from 2011 to 2023. During the entire period, the global Moran *I* values remained positive, ranging from 0.23 to 0.31, indicating a stable pattern of clustering and positive spatial autocorrelation. Local Moran *I* analysis revealed stable low-low clustering of lung cancer PBI in southwestern China, notably in Guangxi. No significant high-high clusters, high-low outliers, or low-high outliers were identified.

**Table 2.** Spatial aggregation of the per capita Baidu Index for lung cancer, from 2011 to 2023. Provinces with nonsignificant local Moran *I* are not listed.

Year	Global Moran *I*		Local Moran *I* (Low-Low cluster)
	Moran *I*	*P* value	
2011	0.27	.03	Guangxi, Guizhou, Shandong
2012	0.27	.03	Guangxi, Guizhou, Hunan
2013	0.31	.01	Guangxi, Guizhou, Shandong
2014	0.30	.02	Guangxi, Guizhou
2015	0.28	.02	Guangxi, Guizhou
2016	0.27	.03	Guangxi, Shandong
2017	0.26	.03	Guangxi
2018	0.25	.03	Guangxi, Guizhou
2019	0.23	.046	Guangxi, Guizhou
2020	0.23	.04	Guangxi, Shandong
2021	0.25	.03	Guangxi, Shandong
2022	0.26	.03	Guangxi, Shandong
2023	0.27	.02	Guangxi, Shandong

### Explanatory Factors

Preliminary Spearman rank correlation analysis suggested that, in certain years, urbanization rate, average years of education per capita, and GDP per capita were positively correlated with PBI of lung cancer, whereas old-age dependency ratio and male-female ratio showed negative correlations (Figure S6 in Multimedia Appendix 1 [26-33]). Table 3 displays the SPDM results for the PBI of lung cancer. Higher GDP per capita and education levels were positively correlated with PBI of lung cancer, whereas the urbanization rate was negatively correlated. Significant positive spatial spillover effects were observed for the PBI of lung cancer (spatial ρ=0.380, *P*<.001). The old-age dependency ratio, GDP per capita, and average years of education per capita were significantly associated with the PBI for lung cancer in neighboring regions. Spillover effects were further examined by decomposing the SPDM into direct and indirect effects using partial differential decomposition. The decomposition of direct and indirect effects revealed that GDP per capita and average years of education per capita were positively correlated with the PBI for lung cancer locally and in adjacent regions, while urbanization was significantly negatively associated with PBI in both spatial contexts.

**Table 3.** Results of the spatial panel Durbin model examining the associations between explanatory variables and per capita Baidu Index.

Variable	Model results (95% CI)		Decomposition of spatial effects (95% CI)		
	Coefficient	Spatial spillover (Wx)	Direct effect	Indirect effect	Total effect
Spatial autoregressive coefficient (ρ)	0.380 (0.272 to 0.489)^a^	**—** ^b^	**—**	**—**	**—**
Male-female ratio	−0.007 (−0.016 to 0.001)	−0.009 (−0.026 to 0.008)	−0.008 (−0.016 to 0.001)	−0.004 (−0.01 to 0.001)	−0.012 (−0.026 to 0.002)
Old-age dependency ratio	0 (−0.001 to 0.001)	−0.003 (−0.006 to −0.001)^c^	0 (−0.001 to 0.001)	0 (−0.001 to 0.001)	0 (−0.002 to 0.002)
Urbanization rate	−0.006 (−0.007 to −0.004)^a^	0 (−0.003 to 0.003)	−0.006 (−0.007 to−0.005)^a^	−0.003 (−0.005 to −0.002)^a^	−0.009 (−0.012 to −0.007)^a^
Ln (GDP^d^ per capita)^e^	0.039 (0.014 to 0.065)^c^	−0.1 (−0.148 to −0.052)^a^	0.041 (0.015 to 0.068)^c^	0.023 (0.007 to 0.045)^f^	0.064 (0.022 to 0.11)^c^
Average years of education per capita	0.027 (0.018 to 0.036)^a^	−0.025 (−0.045 to −0.004)^f^	0.029 (0.02 to 0.038)^a^	0.016 (0.009 to 0.027)^c^	0.045 (0.029 to 0.062)^a^

a Statistically significant at .001.

b Not applicable.

c Statistically significant at .01.

d GDP: gross domestic product.

e Ln (GDP per capita) indicates the logarithm of GDP per capita.

f Statistically significant at .05.

### Topic Modeling

**Figure 4.**
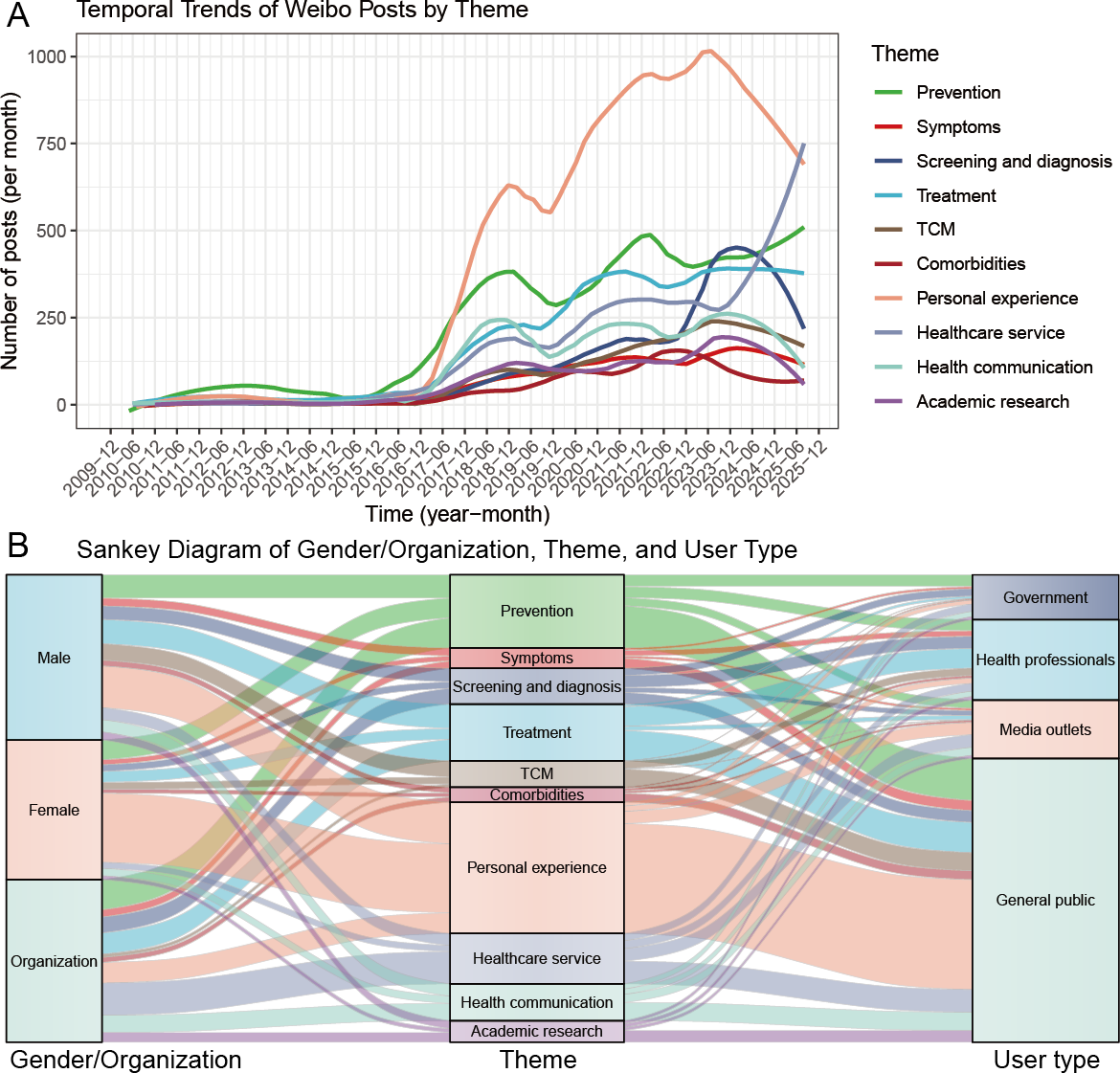
Temporal trends of Weibo posts by theme (A) and Sankey diagram of gender/organization, theme, and user type (B). TCM: traditional Chinese medicine.

Topic modeling of 268,756 lung cancer-related Weibo posts yielded 50 latent topics. Table S7 in Multimedia Appendix 1 [26-33] presents the 50 topics together with the top 20 unique keywords for each topic, which were translated into English by a native Chinese-speaking researcher. Table 4 illustrates 10 main lung cancer-related themes, including health care service (28,902/268,756, 10.75%), treatment (32,609/268,756, 12.13%), prevention (42,096/268,756, 15.66%), traditional Chinese medicine (TCM) (15,112/268,756, 5.62%), personal experience (75,478/268,756, 28.08%), comorbidities (8656/268,756, 3.22%), screening and diagnosis (20,548/268,756, 7.65%), health communication (21,194/268,756, 7.89%), academic research (12,442/268,756, 4.63%), and symptoms (11,719/268,756, 4.36%). As shown in Figure 4A, personal experience emerged as the most prevalent lung cancer theme, followed by prevention, treatment, and health care service. Notably, starting around late 2023, the number of Weibo posts related to personal experience and screening and diagnosis declined markedly, while on health care service rose rapidly, overtaking personal experience in August 2025 as the dominant theme. Figure 4 illustrates the distribution of themes by sex/organization and user type. Female users posted more personal experience–related content and fewer posts on TCM than male users, while the vast majority of posts on health care service were made by institutional accounts. When stratified by user type, government accounts most frequently addressed prevention, health professionals emphasized treatment, media outlets focused on health care service and personal experience, and the general public predominantly shared personal experiences.

**Table 4.** Themes identified from the 50 lung cancer-related topics on Weibo.

Frame	Posts (n=268,756), n (%)	Description	Subtopics	Representative keywords
Health care service	28,902 (10.75)	Hospital services, insurance coverage, community care, and medical technologies	1, 15, 28, 29, 35, 45, 48	Service, genetic testing, consultation, medical insurance, cost, technology, precision, AI^a^, hospital, expert, tumor, professor, product, market, project, health, hygiene, community
Treatment	32,609 (12.13)	Clinical interventions including surgery, chemotherapy, radiotherapy, targeted therapy, and immunotherapy	2, 10, 14, 22, 33, 47	Treatment, patient, combination, first-line, chemotherapy, PD-1, radiotherapy, tumor, drug, survival, immunotherapy, side effect, targeted drug, surgery, thoracic surgery, pathology
Prevention	42,096 (15.66)	Lifestyle and environmental factors related to lung cancer risk reduction	3, 5, 12, 17, 18, 41, 49	Prevention, health, exercise, lifestyle, sleep, reduce, risk, immunity, diet, stress, smoking, cooking oil fume, pollution, air, indoor, environment, food products, fat, obesity, nutrition
TCM^b^	15,112 (5.62)	Use of Chinese herbal medicine, syndrome differentiation, and health preservation practices	4, 24, 26	Traditional Chinese medicine, Chinese herbal medicine, anti-cancer, effect, lung cancer, relief, licorice, pinellia, resolve phlegm, astragalus, prescription, poria, health preservation
Personal experience	75,478 (28.08)	Patient and family narratives of illness, treatment, and caregiving	6, 7, 11, 13, 23, 30, 31, 34, 37, 46	Lung cancer, one year, detected, surgery, hope, wife, last year, family, anti-cancer, friend, aunt, hospitalization, elderly, evening, feeling, several days, go home, death, passing away, donation
Comorbidities	8656 (3.22)	Coexisting conditions such as cardiovascular disease, diabetes, and infections	8, 9, 44	Disease, diabetes, health, hypertension, cardiovascular, heart disease, elderly, blood pressure, prevention, blood glucose, skin, COVID-19, pneumonia, patient, vaccine, fever, vein, hormone
Screening and diagnosis	20,548 (7.65)	Early detection through imaging, physical examination, tumor markers, and clinical vigilance	16, 21, 27	Examination, screening, CT^c^, recommendation, testing, detection,tumor marker, ultrasound, age 50, high-risk population, diagnosis, chest radiograph, dosage, health, pulmonary nodule
Health communication	21,194 (7.89)	Public communication and education through media, experts, and online platforms	19, 32, 39, 40	China, world, lung cancer, science, life expectancy, society, mortality rate, doctor, hospital, examination, recommendation, answer, consultation, science popularization, trend
Academic research	12,442 (4.63)	Scientific studies on lung cancer	20, 36, 38, 42, 43	Research, discovery, cell, immunity, tumor, cancer cell, function, inhibition, system, growth, mechanism, gene, scientist, this study, antibody, direction, related, strain, product, antigen
Symptoms	11,719 (4.36)	Bodily signs and clinical manifestations	25, 50	Symptom, pain, manifestation, signal, body, vigilance, cause, situation, abnormal, nerve, lump, examination, site, persistent, skin, hoarseness, compression, ache, joint, cough, phlegm

a AI: artificial intelligence.

b TCM: traditional Chinese medicine.

c CT: computed tomography.

### Sentiment Analysis

**Figure 5.**
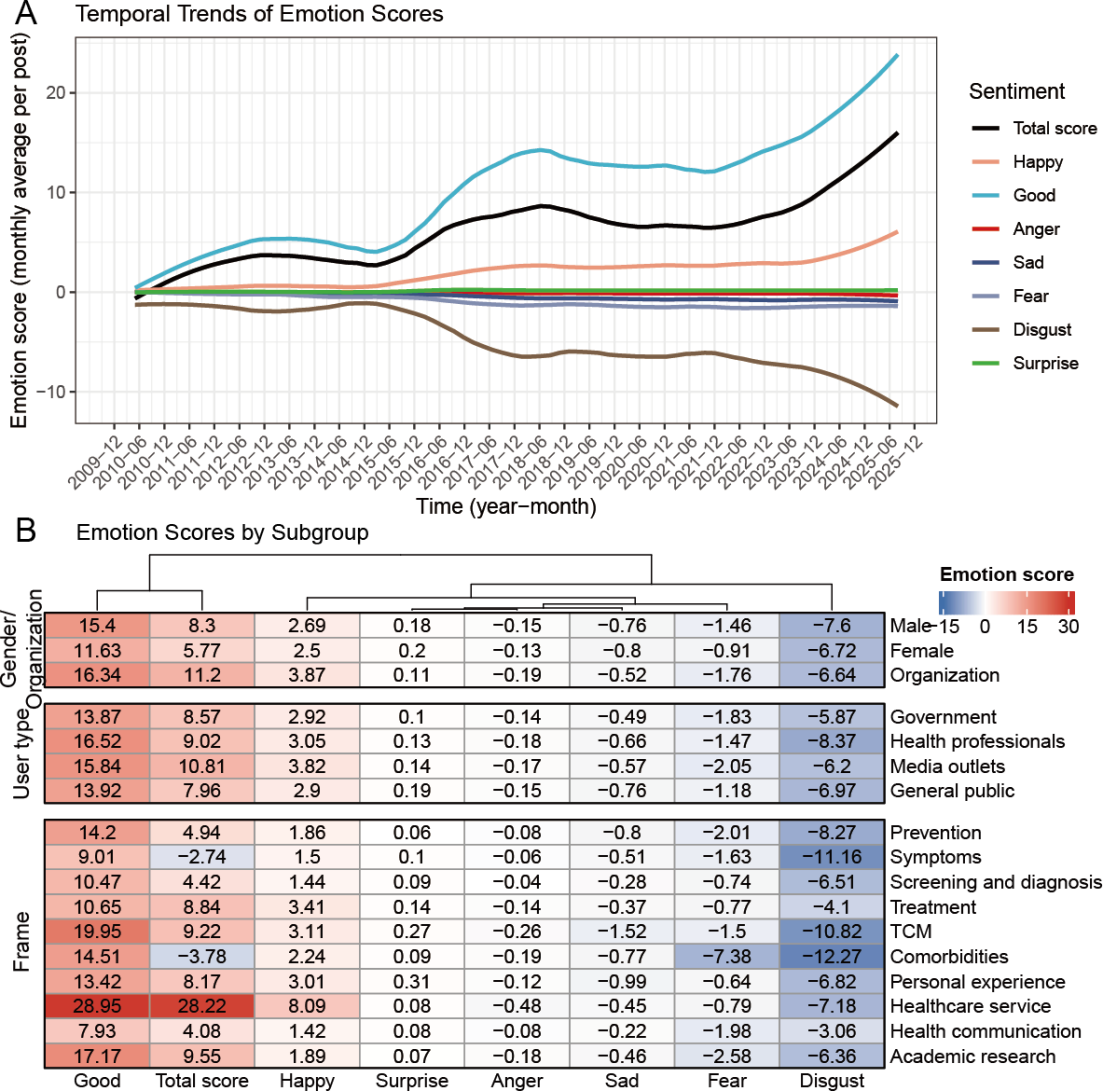
Temporal trends of emotion scores by sentiment category (A) and emotion scores by sex/organization, user type, and theme (B). TCM: traditional Chinese medicine.

Figure 5A depicts the temporal trends in emotion scores of Weibo posts related to lung cancer. In the sentiment scores, the sign indicates emotional polarity (positive or negative), whereas the absolute value reflects the intensity of the emotion. From 2010 to 2025, the total emotion score was consistently positive and increased gradually with fluctuation. Among the 7 sentiment categories, “Good” and “Disgust” were the dominant positive and negative emotions, respectively, both of which increased in intensity over time. Moreover, “Happy” remained relatively stable, and other emotions were expressed only at low levels. Further subgroup-specific results of the sentiment analysis are shown in Figure 5, Figure S7 and Table S8 in Multimedia Appendix 1 [26-33]. Sex/organization comparisons showed higher sentiment scores among institutional accounts, followed by male and then female users. By user type, the order was media outlets, health professionals, government, and the general public. Thematic analysis revealed health care services as the most positively scored theme, followed at a distance by academic research and TCM, whereas comorbidities and symptoms scored negatively. Moreover, sentiment trajectories varied across subgroups, with government, media outlets, prevention, personal experience, symptoms, comorbidities, and academic research exhibiting notable downward trends in recent years.

## Discussion

### Principal Findings

This study comprehensively examined public attention, perceptions, and sentiment toward lung cancer in China by integrating search engine and social media data. Temporally, search engine data showed that public attention peaked in 2019 and declined thereafter, while Weibo discussions have remained relatively stable since 2022. Spatial heterogeneity and autocorrelation were observed, with low-low clusters concentrated in the southwestern region, particularly Guangxi. GDP per capita and average years of education were positively associated with PBI, whereas urbanization rate showed a negative correlation. Topic modeling revealed a temporal shift in themes, with personal experiences initially predominant and health care services emerging as the leading theme in 2025. Overall sentiment was generally positive, mainly characterized by “Good” and “Disgust,” with variations across years, sex/organization, and user types. These findings provide a foundation for future research and health communication strategies.

To the best of our knowledge, this study is the first to offer a dual-perspective analysis that captures both macro-level public engagement and micro-level social discourse regarding lung cancer in China. Existing research in English-language digital contexts has primarily focused on how lung cancer discourse manifests on platforms such as Twitter and Facebook, revealing distinct communication dynamics and thematic contents [40,41]. However, China’s socioeconomic and cultural context shapes digital health communication in distinct ways. While Chinese social platforms such as Weibo align with some global patterns as a space for personal narrative expression, it is also deeply embedded in China’s local health discourse and cultural dissemination. TCM, for example, frequently appeared in user narratives as both a therapeutic option and a cultural lens [42]. Examining Weibo’s health discourse, this study provides a contextual insight into how lung cancer is communicated in China’s digital sphere.

The temporal trends observed in public attention to lung cancer have followed an inverted U-shaped trajectory over the past 15 years. The sharp rise in BI before its 2019 peak may reflect increased public concern promoted by policy actions, media coverage, or celebrity illness disclosures. Such surges align with event-driven information-seeking, characteristic of public health crises, where perceived threats heighten public attention [43]. The post-2019 decline, however, indicates reduced public attention, potentially due to the normalization of lung cancer or a diversion of public focus to emerging issues such as COVID-19 [42]. In addition, changes in search algorithms and the growing use of other web platforms may also have influenced the observed trends in public attention. Notably, the continued growth of Weibo activity until 2022 may imply a shift in public discourse from active information-seeking to more passive or peer-oriented engagement. The observed divergence suggests a functional distinction: search engines provide immediate information access, whereas social media enable ongoing, socially embedded discourse [41,44]. Collectively, these patterns indicate that platform-specific strategies are essential for sustaining public awareness and reinforcing lung cancer-related health communication.

Spatial disparities in public attention toward lung cancer were evident across provincial-level regions in China. Epidemiological data from 2021 demonstrated that the age-standardized incidence and mortality rates of lung cancer were highest in northeast Chinese provinces, particularly in Heilongjiang, Tianjin, and Liaoning, while Xinjiang, Gansu, and Tibet reported the lowest incidence rates [45]. However, the regional distribution of lung cancer burden diverges notably from that of BI or PBI. The high BI for lung cancer in eastern and southeastern coastal regions may reflect their more developed socioeconomic structures and higher population density [26,46]. In contrast to BI, PBI clusters emerged in regions such as Beijing, Hainan, Ningxia, Qinghai, Shanghai, and Tianjin, indicating that public attention may be shaped more by contextual factors (eg, media coverage, health education programs, or local policy) than by actual disease burden alone. This pattern may also be partly explained by the high concentration of top-tier medical resources in these regions, which may help sustain public awareness and attention to lung cancer. In addition, the low-low clustering observed in southwestern China, particularly in Guangxi, may indicate geographically concentrated areas of relatively limited engagement with lung cancer-related information, potentially reflecting regional disparities in health information access, awareness, or digital health literacy. In these regions, public health authorities may consider prioritizing targeted offline awareness campaigns for lung cancer screening, alongside strengthening digital health literacy programs to improve access to and engagement with relevant health information. The factors correlated with web-based attention to lung cancer offer further insight into the interpretation. The association of higher GDP per capita and average years of education per capita suggests that provinces with greater socioeconomic resources tended to exhibit higher levels of lung cancer-related web activity. At the macro level, this pattern may be associated with broader regional differences in digital infrastructure and access to health-related information [47,48]. Conversely, the negative association with urbanization rate, while unexpected, may reflect greater diversification of health information channels and a lower aggregate level of curiosity-driven search activity in more highly urbanized regions [26]. This interpretation should be treated with caution, as the VIF of the urbanization rate was 6.85, indicating moderate multicollinearity, and the negative coefficient may be partly affected by statistical collinearity. It is also worth noting that these associations should be interpreted at the regional level and do not represent individual information-seeking intentions. Moreover, the spatial panel econometric model confirmed both direct and spillover effects. For example, provinces with higher education levels were associated with higher local PBI and showed positive spatial associations with PBI in neighboring regions, possibly reflecting knowledge diffusion and cross-regional interactions. These findings highlight the importance of accounting for socioeconomic variations and spatial interdependence when interpreting digital engagement with lung cancer information.

Topic modeling and thematic analysis revealed micro-level discourse on lung cancer among Chinese Weibo users. Since late 2023, posts related to health care services have grown rapidly, surpassing personal experience by mid-2025. This transition reflects a shift from personal narratives to system-level concerns related to health care accessibility, affordability, and quality. At the same time, this increase may also partly reflect the growing presence of institutional and commercial actors in the web-based health information space. Other persistent themes included prevention, treatment, screening, and diagnosis, indicating a sustained public attention to early detection and clinical management. Besides, the sustained presence of TCM underscores the culturally embedded nature of discourse in Chinese digital health contexts [42]. Temporal trends in theme prevalence also aligned with policy shifts and media events. For example, the outbreak of COVID-19 in December 2019 may have heightened public awareness of respiratory diseases, triggering a reversal in the trajectory of personal-experience posts from decline to rise [42,49,50]. Meanwhile, the recent decline in content related to screening and diagnosis suggests a potential misalignment between public discourse and long-term health priorities. These findings emphasize that public health communication should integrate patient-centered needs with systemic issues and actively promote preventive strategies.

Sentiment analysis provided an additional perspective on web-based discourse about lung cancer. Overall, sentiment remained positive between 2010 and 2025, with an upward trend despite fluctuations, indicating a generally optimistic and supportive tone. Emotional expression was concentrated within a narrow spectrum, with “Good” representing the primary positive category and “Disgust” the principal negative one. Both increased in intensity over time, illustrating an emotional landscape in which respect, praise, trust, and affection coexisted with irritation, detestation, criticism, jealousy, and suspicion [38]. Subgroup differences highlighted how sex/organization, communicative roles, and thematic focus shape sentiment in web-based discourse. Higher scores among institutional accounts and media outlets suggested deliberate efforts to frame discourse positively, while lower scores from females may reflect both social vulnerability and a tendency to express distress more directly. Similarly, positive sentiment in health care service posts and negative content on comorbidities and symptoms underscored the dual role of digital platforms as spaces for health communication and outlets for distress.

### Limitations

Several limitations should be acknowledged in this study. First, data accessible via the BI platform is constrained to predefined search terms. Consequently, infrequently used or rare expressions may be excluded from trend analyses. Second, the Weibo user base is not representative of the broader Chinese population. Its users tend to be younger, reside in urban areas, and possess higher levels of digital literacy, potentially introducing bias into the findings. Therefore, given that respiratory tract cancers disproportionately affect older males, the observed patterns should be interpreted as reflecting the perceptions and expressions of digitally active users rather than those of the primary high-risk population directly affected by the disease. Moreover, this study adopts an observational and descriptive design. Although associations between explanatory variables and PBI were observed, causal inferences cannot be made. The lack of reliable provincial data on internet users prevented adjustment for internet penetration, potentially introducing regional denominator bias. Unmeasured confounders, such as lung cancer incidence, air pollution, and local public policy, may also influence the patterns of web-based discourse. In addition, although COVID-19 may have influenced search behavior and spatial spillover patterns, pandemic-specific controls were not included due to data limitations, and future research with more granular indicators is warranted. Furthermore, lexicon-based sentiment analysis methods exhibit inherent limitations. Specifically, challenges such as polysemy, context-dependent sarcasm, and culturally specific meanings may undermine the reliability of emotion classification. Finally, this study focused exclusively on public perceptions of lung cancer within Mainland China. To improve cross-cultural applicability and spatial resolution of future research, international comparative studies and more fine-grained spatial analyses are warranted.

### Conclusions

This study provides a comprehensive examination of public perceptions of lung cancer in Mainland China by integrating search engine and social media data. The findings showed that public attention peaked in 2019 and declined thereafter, while Weibo discussions remained stable since 2022. Spatial heterogeneity and low-low clusters were observed, such as in Guangxi. PBI was positively associated with GDP per capita and average educational attainment but negatively associated with the urbanization rate. Topic modeling revealed a thematic shift from personal experience to health care service by 2025, and sentiment analysis indicated an overall positive tone driven by “Good” and “Disgust.” These findings contribute to understanding lung cancer discourse in China’s digital landscape and provide evidence for improving targeted health communication.

## Supplementary material

10.2196/85058 Multimedia Appendix 1 Supplementary methods, figures, and tables related to the study.

## Abbreviations

BI: Baidu Index
EAPC: estimated annual percentage change
GDP: gross domestic product
LDA: latent Dirichlet allocation
PBI: per capita Baidu Index
SPDM: spatial panel Durbin model
TCM: traditional Chinese medicine
VIF: variance inflation factor
