# Supplementary material for: Understanding Public Perceptions of Lung Cancer in China: Infodemiology Study of Baidu Index and Weibo Posts
Source: J Med Internet Res. 2026 May 5;28:e85058. doi: 10.2196/85058 (PMC13143159; doi:10.2196/85058)
Supplement: Multimedia Appendix 1 [file jmir-v28-e85058-s001.docx]

***Supplementary Material***

**Understanding Public Perceptions of Lung Cancer in China: Insights From Search Engine and Social Media Data**

Contents

[**Supplementary Methods** 3](#_Toc224656830)

[**Supplementary Figure S1.** Spatiotemporal patterns of explanatory variables among provincial-level regions in China. Abbreviations: EAPC, estimated annual percent change; GDP, gross domestic product. 6](#_Toc224656831)

[**Supplementary Figure S2.** Selection process of panel econometric model. Abbreviations: PBI, per capita Baidu index; LM, Lagrange multiplier; RLM, robust Lagrange multiplier; SEM, spatial error model; SDM, spatial Durbin model; SAR, spatial regression model; LR, likelihood ratio. 7](#_Toc224656832)

[**Supplementary Figure S3.** Calculating different metrics to estimate optimal number of topics for LDA models. Abbreviations: LDA, latent Dirichlet allocation. 8](#_Toc224656833)

[**Supplementary Figure S4.** Temporal trends of BI for lung cancer among provincial-level regions in China, from 2011 to 2025. Monthly average values were reported, and LOESS along with GLM models were applied to capture more detailed trends. Abbreviations: BI, Baidu index; LOESS, locally weighted regression; GLM, generalized linear model. 9](#_Toc224656834)

[**Supplementary Figure S5.** Global and local spatial autocorrelation of PBI for lung cancer among provincial-level regions in China, from 2011 to 2023. Regions with statistically significant local Moran’s I values are indicated on the map (*P < 0.05; **P < 0.01). Abbreviations: PBI, per capita Baidu index. 10](#_Toc224656835)

[**Supplementary Figure S6.** Spearman’s correlation heatmap for the associations between explanatory variables and PBI among provincial-level regions in China, from 2011 to 2023. The Benjamin-Hochberg procedure was applied to control for multiple tests. Abbreviations: PBI, per capita Baidu index; GDP, gross domestic product. 11](#_Toc224656836)

[**Supplementary Figure S7.** Temporal trends of emotion scores for lung cancer in China, by gender/organization, user type, and theme, from 2010 to 2025. Abbreviations: TCM, traditional Chinese medicine. 12](#_Toc224656837)

[**Supplementary Table S1.** Descriptive statistics and fixed-effects panel regression results for explanatory variables across provincial-level regions in China, 2011-2023. 13](#_Toc224656838)

[**Supplementary Table S2.** The definition of the spatial neighbours of each province-level region in China. 14](#_Toc224656839)

[**Supplementary Table S3.** Results of variance inflation factor test for explanatory variables. 16](#_Toc224656840)

[**Supplementary Table S4.** Results of the spatial panel econometric model selection test. 17](#_Toc224656841)

[**Supplementary Table S5.** Emotion Taxonomy and Example Lexemes of DLUT-Emotion Ontology. 18](#_Toc224656842)

[**Supplementary Table S6.** The concentration ratio of the daily average Baidu index for lung cancer, 2011-2023. 19](#_Toc224656843)

[**Supplementary Table S7.** Fifty lung cancer–related topics on Weibo with top 20 keywords and manually annotated themes. 20](#_Toc224656844)

[**Supplementary Table S8.** Sentiment analysis results for Weibo posts on lung cancer by subgroup. 24](#_Toc224656845)

# **Supplementary Methods**

**1. Data Sources**

1.1 Text Preprocessing

Text preprocessing was performed to clean lung cancer–related Weibo posts prior to topic modeling using latent Dirichlet allocation (LDA) and sentiment analysis ^1–3^. Specifically, contents embedded within specific tag formats (e.g., “（）”, “[]”, “《》”, “##”, and “<>”) were deleted. Usernames (e.g., “@Username”) and hyperlinks (e.g., “http://weibo.com”) were also stripped from the text to reduce noise. In addition, all extraneous symbols and punctuation marks (e.g., “!”, “//”) were eliminated, and all spaces were removed. Following text cleaning, Chinese word segmentation was performed using the JiebaR package (version 0.11). To further refine the corpus, common stopwords were filtered using several widely recognized Chinese stopword dictionaries, including the HIT stopwords list (developed by Harbin Institute of Technology), Baidu stopwords (provided by Baidu Inc.), and SCU stopwords (created by the Machine Intelligence Laboratory at Sichuan University) ^4^. These dictionaries helped exclude high-frequency functional words that carry little semantic value in natural language processing (e.g., equivalents of “the,” “a,” “an,” and “in”). Finally, tokenization was carried out to segment the cleaned text into semantically meaningful units for subsequent analysis.

1.2 Explanatory Variables

The data used in this study were primarily obtained from *the China Statistical Yearbook* and *the Sixth and Seventh National Population Censuses*. The dataset covers the period from 2011 to 2023 and includes information on the educational attainment of the population aged six years and above across 31 provinces, autonomous regions, and municipalities in mainland China (excluding Hong Kong, Macao, and Taiwan). Following the classification established in previous research ^5^, the average number of years of schooling for individuals aged six and above in each province is denoted as follows:

$$\begin{aligned} y_{\text{school }}=\sum_{i=0}^{4} y_{i} \#\left( 1 \right) \end{aligned}$$

$$\begin{aligned} y_{0}=\frac{p_{0}}{p_{6plus}}\times0,\quad y_{1}=\frac{p_{1}}{p_{6plus}}\times6,\quad y_{2}=\frac{p_{2}}{p_{6plus}}\times9,\quad y_{3}=\frac{p_{3}}{p_{6plus}}\times12,\quad y_{4}=\frac{p_{4}}{p_{6plus}}\times16\#\left( 2 \right) \end{aligned}$$

where $yi$ represents the contribution to the average schooling years from group i, p_i_ refers to the population of group $i$, and $P_{6plus}$ is the total population. The weights are assigned according to the official schooling duration for each education level: 0 years for no formal schooling, 6 years for primary school, 9 years for junior secondary, 12 years for senior secondary, and 16 years for tertiary education and above.

**2. Statistical Analysis**

2.1 Temporal Aggregation Analysis

Temporal trends of provincial BI and PBI from 2011 to 2023 were assessed through estimated annual percent change (EAPC). The EAPC was estimated using a linear regression model, defined by the following equation:

$$\begin{aligned} y=\alpha+\beta x+\varepsilon\#\left( 3 \right) \end{aligned}$$

$$\begin{aligned} EAPC=100\times\left( \exp\left( \beta\right)-1 \right)\#\left( 4 \right) \end{aligned}$$

where y represents the natural logarithm of the BI/PBI and x denotes the calendar year.

To assess temporal aggregation of the BI for lung cancer, we applied the concentration ratio method ^6^. The index M ranges from 0 to 1; larger values denote stronger aggregation, while values below 0.3 indicate a relatively uniform distribution over time. The formula is expressed as follows:

$$\begin{aligned} R_{x}=\frac{r_{2}+r_{6}-r_{8}-r_{12}}{2}+\frac{\sqrt{3}\left( r_{3}+r_{5}-r_{9}-r_{11} \right)}{2}+\left( r_{4}-r_{10} \right)\#\left( 5 \right) \end{aligned}$$

$$\begin{aligned} R_{y}=\frac{r_{3}-r_{5}-r_{9}+r_{11}}{2}+\frac{\sqrt{3}\left( r_{2}-r_{6}-r_{8}+r_{12} \right)}{2}+\left( r_{1}-r_{7} \right)\#\left( 6 \right) \end{aligned}$$

$$\begin{aligned} M=\sqrt{R_{x}^{2}+R_{y}^{2}}\#\left( 7 \right) \end{aligned}$$

where R denotes the degree of dispersion, and r indicates the proportion of searches in a given month relative to the total annual search volume.

2.2 Spatial Panel Econometric Model Description

The main classical spatial panel econometric models, including spatial autoregressive model (SAR), spatial error model (SEM), and spatial Durbin model (SDM), differ in how they model spatial dependence ^7,8^. SAR captures spatial autocorrelation in the dependent variable through the inclusion of spatial lags of the regressors. SEM, in contrast, models spatial dependence via the error structure. SDM combines both spatial lag and error dependence, offering a more comprehensive framework. The mathematical structure of these models is outlined as follows:

$$\begin{aligned} y_{it}=\alpha+\rho w_{ij}y_{it}+\beta_{1}x_{it}+\beta_{2}w_{ij}x_{it}+\varepsilon_{it}\#\left( 6 \right) \end{aligned}$$

$$\begin{aligned} \varepsilon_{it}=\lambda w_{ij}\varepsilon_{it}+\mu_{it}\#\left( 7 \right) \end{aligned}$$

Here, $y_{it}$ denotes the dependent variable for unit $i$ at time $t$, while $x_{it}$ represents the explanatory variable. The term α is the intercept term, and ρ captures the spatial autoregressive coefficient associated with the spatial lag of the dependent variable, where $w_{ij}$ is the spatial weight matrix defining the spatial relationship between units $i$ and $j$. The coefficients β_1_ and β_2_ correspond to the direct effect of $x_{it}$ and its spatial lag $w_{ij}x_{it}$, respectively. ε denotes the spatially autocorrelated error term, λ is the spatial error coefficient, and $\mu_{it}$ is the independent and identically distributed disturbance term with zero mean and constant variance. The model simplifies to the SAR when λ=0 and β_2_ =0; to the SEM when ρ=0 and β_2_ =0; and to the SDM when λ=0.

**References:**

1. Zheng S, Wang J, Sun C, Zhang X, Kahn ME. Air pollution lowers Chinese urbanites’ expressed happiness on social media. *Nat Hum Behav*. 2019;3(3):237-243. doi:10.1038/s41562-018-0521-2

2. Kong D, Chen A, Zhang J, et al. Public discourse and sentiment toward dementia on Chinese social media: Machine learning analysis of weibo posts. *J Med Internet Res*. 2022;24(9):e39805. doi:10.2196/39805

3. Chen X, Yik M. The emotional anatomy of the wuhan lockdown: Sentiment analysis using weibo data. *JMIR Form Res*. 2022;6(11):e37698. doi:10.2196/37698

4. Guan Q, Deng S, Wang H. Chinese Stopwords for Text Clustering: A Comparative Study. *Data Analysis and Knowledge Discovery*. 2017;1(03):72-80. doi:CNKI:SUN:XDTQ.0.2017-03-013

5. Zeng Y, Zhou Y, Li K. Inter-Provincial Differences and Trends in Average Years of Education Per Capita. *China Economics of Education Review*. 2024;9(01):104-121. doi:10.19512/j.cnki.issn2096-2088.2024.01.002

6. Zhang Z, Xu H, Pan J, Song F, Chen T. Spatiotemporal characteristics and influential factors of electronic cigarette web-based attention in mainland China: Time series observational study. *J Med Internet Res*. 2025;27:e66446. doi:10.2196/66446

7. Liu C, Nie G. Spatial effects and impact factors of food nitrogen footprint in China based on spatial durbin panel model. *Environ Res*. 2022;204(Pt B):112046. doi:10.1016/j.envres.2021.112046

8. Jin B, Wu Y, Rao CR, Hou L. Estimation and model selection in general spatial dynamic panel data models. *Proc Natl Acad Sci U S A*. 2020;117(10):5235-5241. doi:10.1073/pnas.1917411117

# **Supplementary Figure S1.** Spatiotemporal patterns of explanatory variables among provincial-level regions in China. Abbreviations: EAPC, estimated annual percent change; GDP, gross domestic product.


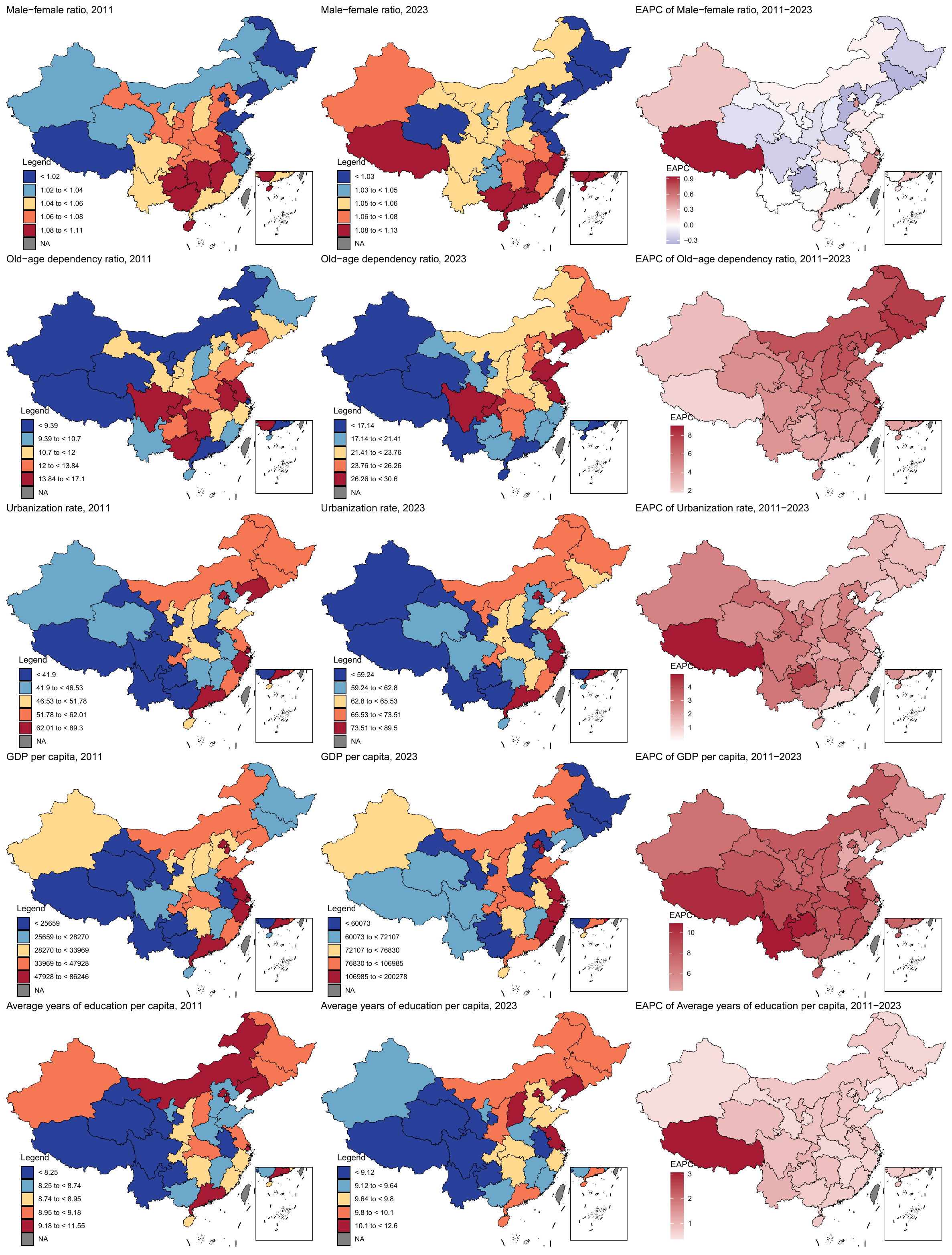


#
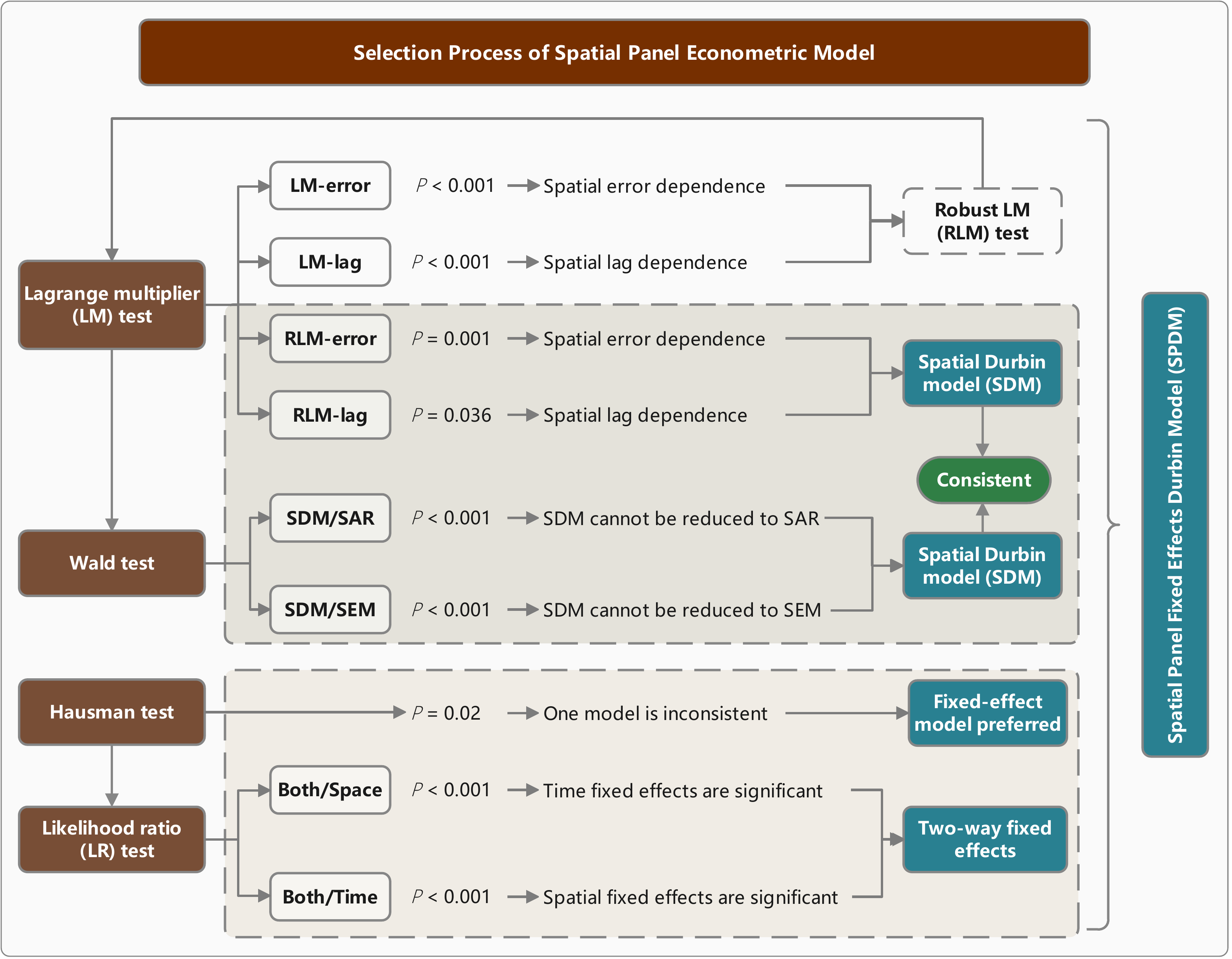
**Supplementary Figure S2.** Selection process of panel econometric model. Abbreviations: PBI, per capita Baidu index; LM, Lagrange multiplier; RLM, robust Lagrange multiplier; SEM, spatial error model; SDM, spatial Durbin model; SAR, spatial regression model; LR, likelihood ratio.

# **Supplementary Figure S3.** Calculating different metrics to estimate optimal number of topics for LDA models. Abbreviations: LDA, latent Dirichlet allocation.


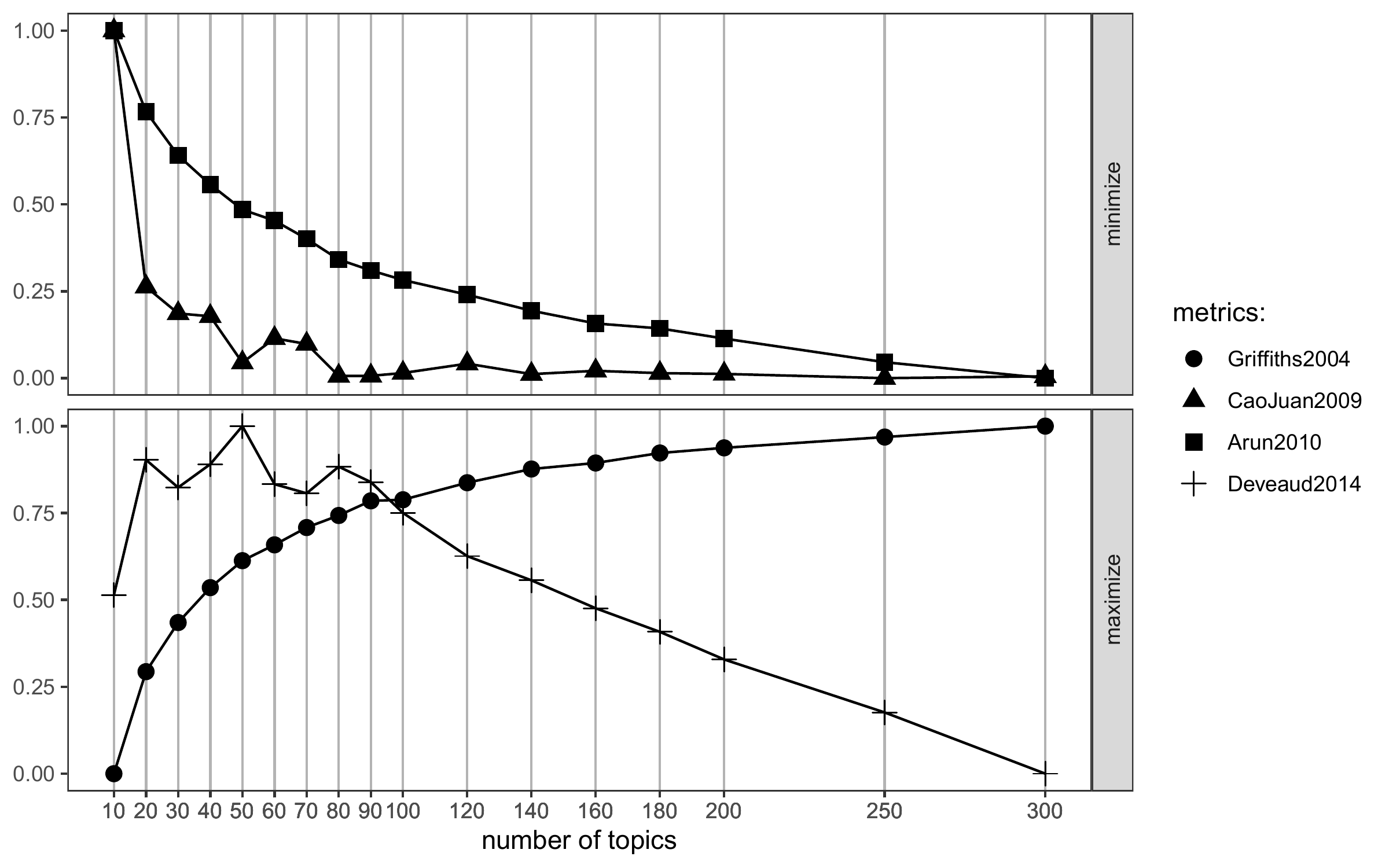


# **Supplementary Figure S4.** Temporal trends of BI for lung cancer among provincial-level regions in China, from 2011 to 2025. Monthly average values were reported, and LOESS along with GLM models were applied to capture more detailed trends. Abbreviations: BI, Baidu index; LOESS, locally weighted regression; GLM, generalized linear model.


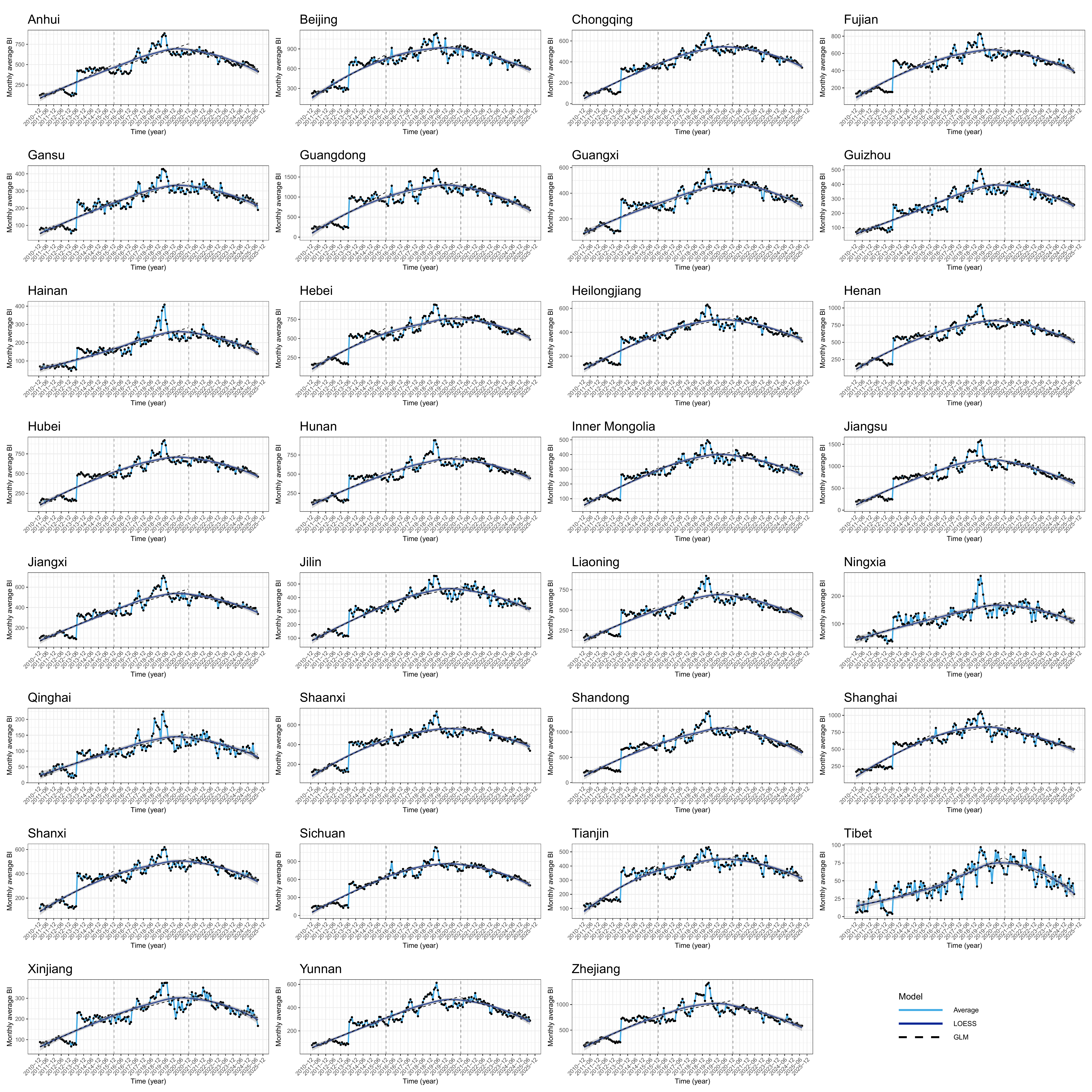


# **Supplementary Figure S5.** Global and local spatial autocorrelation of PBI for lung cancer among provincial-level regions in China, from 2011 to 2023. Regions with statistically significant local Moran’s I values are indicated on the map (*P < 0.05; **P < 0.01). Abbreviations: PBI, per capita Baidu index.


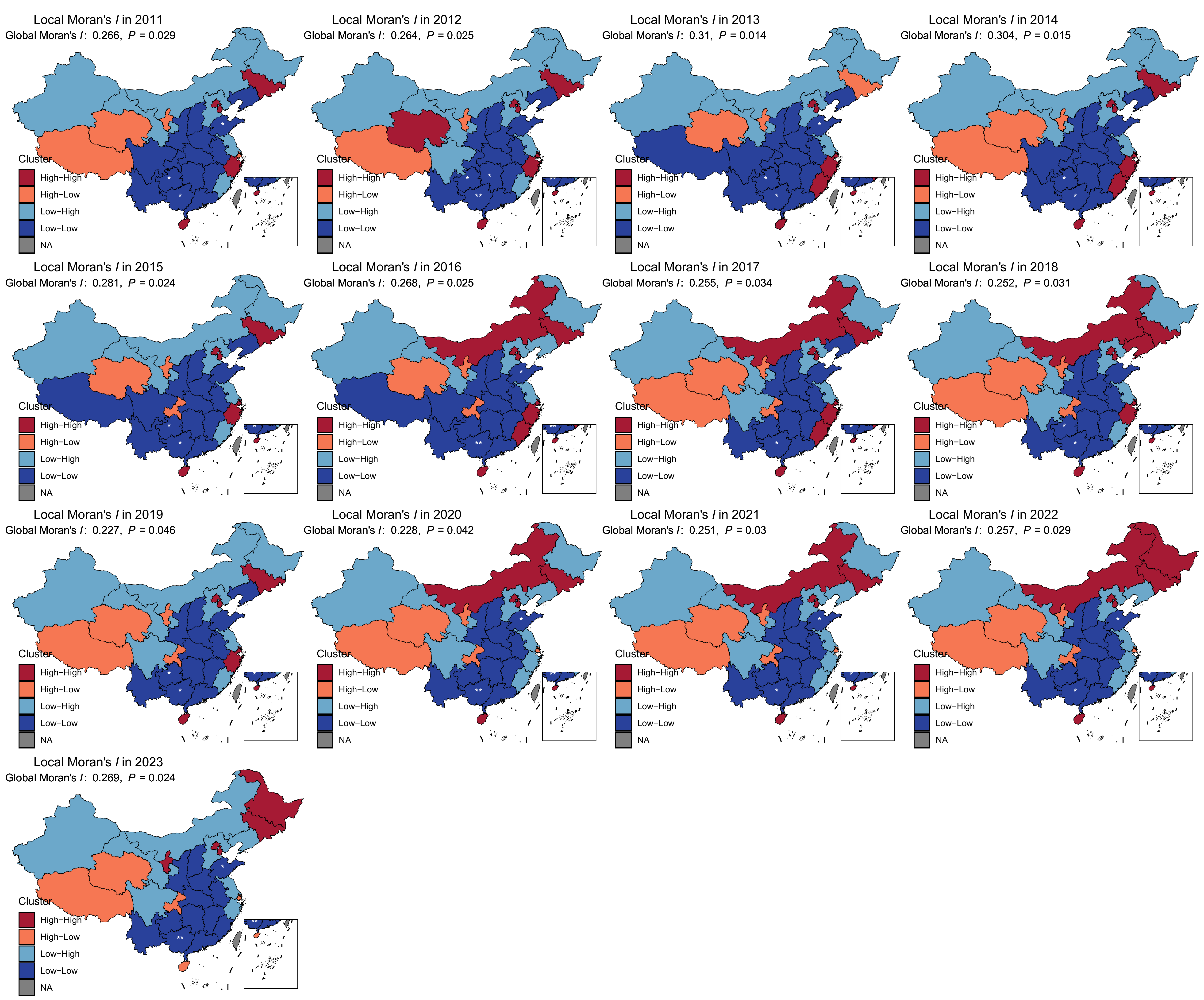


# **Supplementary Figure S6.** Spearman’s correlation heatmap for the associations between explanatory variables and PBI among provincial-level regions in China, from 2011 to 2023. The Benjamin-Hochberg procedure was applied to control for multiple tests. Abbreviations: PBI, per capita Baidu index; GDP, gross domestic product.


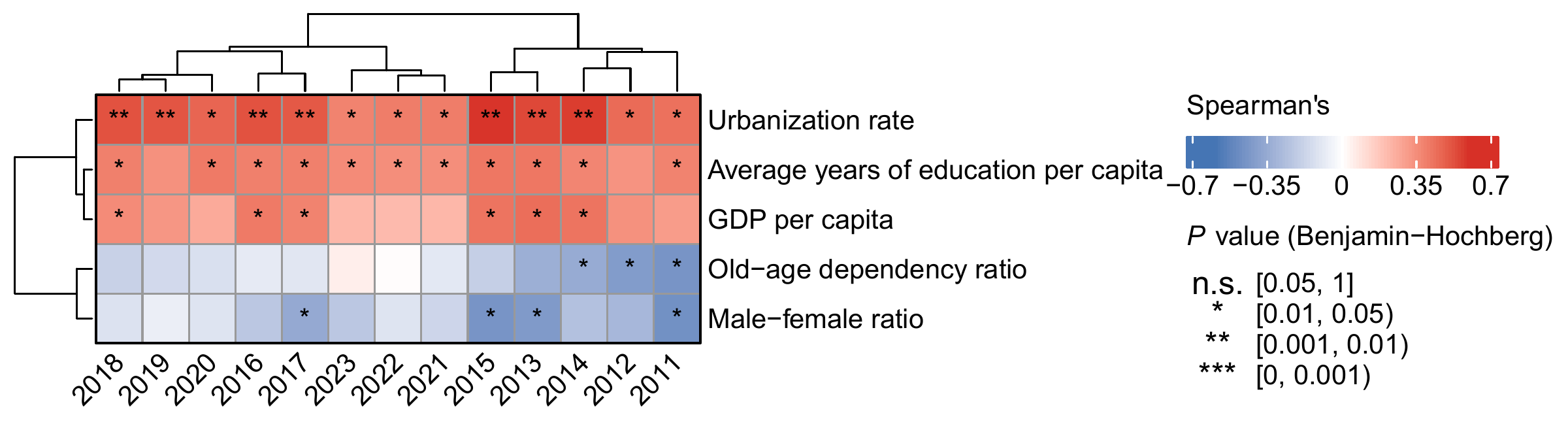


#
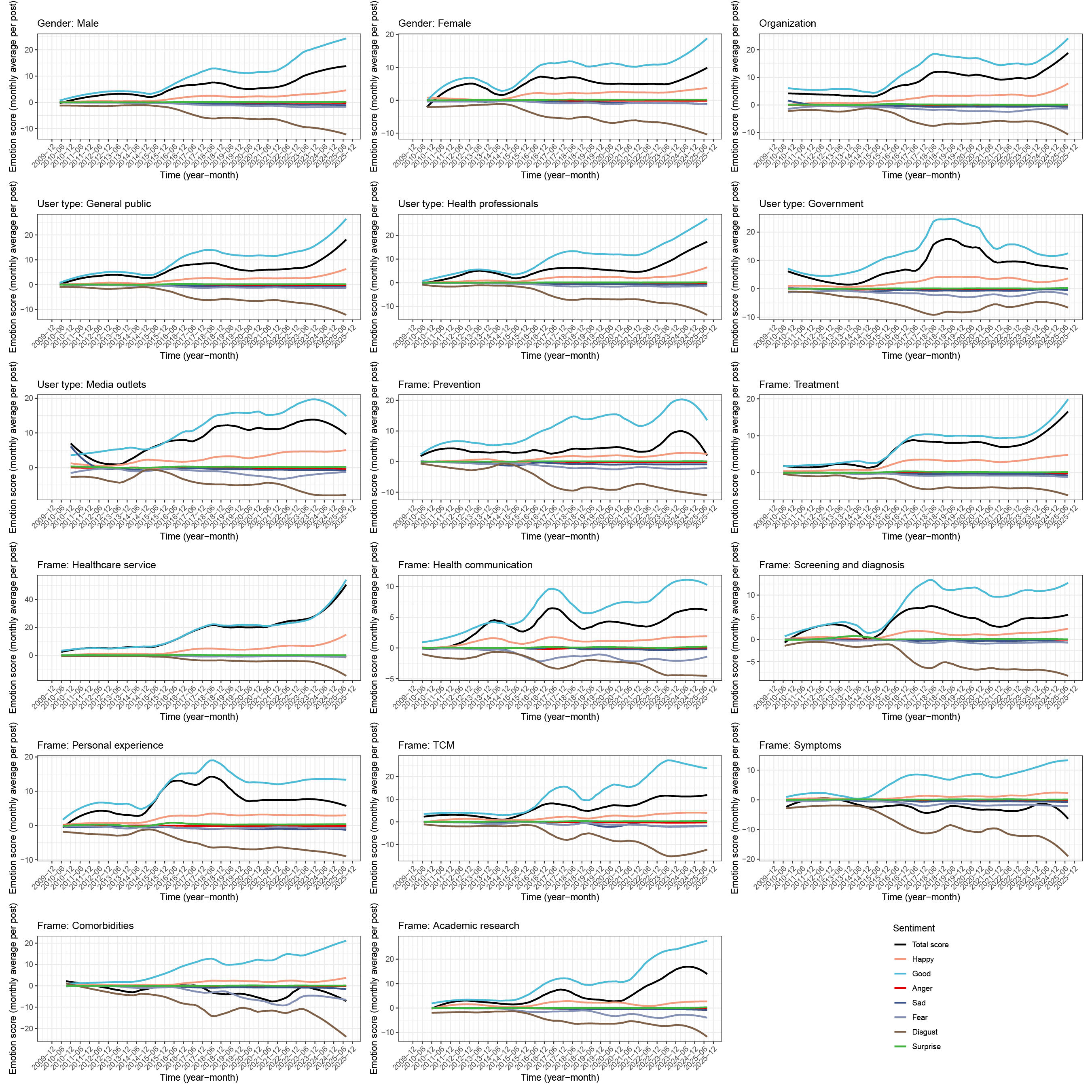
**Supplementary Figure S7.** Temporal trends of emotion scores for lung cancer in China, by gender/organization, user type, and theme, from 2010 to 2025. Abbreviations: TCM, traditional Chinese medicine.

# **Supplementary Table S1.** Descriptive statistics and fixed-effects panel regression results for explanatory variables across provincial-level regions in China, 2011-2023.

| **Characteristic** | **Overall** | **Year** | | | | | | | | | | | | | **Fixed-Effects Panel Regression** | |
| --- | --- | --- | --- | --- | --- | --- | --- | --- | --- | --- | --- | --- | --- | --- | --- | --- |
|  |  | **2011** | **2012** | **2013** | **2014** | **2015** | **2016** | **2017** | **2018** | **2019** | **2020** | **2021** | **2022** | **2023** | **Coefficient** | ***P*** |
| **Male-female ratio (%)** | 1.06 (0.04) | 1.05 (0.03) | 1.05 (0.04) | 1.05 (0.03) | 1.06 (0.04) | 1.07 (0.04) | 1.05 (0.04) | 1.06 (0.03) | 1.06 (0.04) | 1.06 (0.05) | 1.06 (0.03) | 1.06 (0.04) | 1.05 (0.04) | 1.06 (0.03) | -0.03 | < 0.001 |
| **Old-age dependency ratio (%)** | 15.87 (4.94) | 11.54 (2.56) | 12.01 (2.44) | 12.47 (2.54) | 12.95 (2.75) | 13.84 (2.64) | 14.32 (3.10) | 15.13 (3.22) | 15.83 (3.76) | 16.84 (4.00) | 18.83 (4.31) | 19.94 (4.58) | 20.94 (4.92) | 21.73 (5.21) | 0.83 | < 0.001 |
| **Urbanization rate (%)** | 59.68 (12.93) | 52.21 (14.46) | 53.35 (14.25) | 54.65 (13.96) | 55.91 (13.45) | 57.40 (12.85) | 58.89 (12.34) | 60.27 (11.87) | 61.48 (11.58) | 62.54 (11.43) | 63.72 (11.05) | 64.51 (10.80) | 65.00 (10.65) | 65.95 (10.36) | 1.2 | < 0.001 |
| **GDP per capita (Yuan)** | 60,127.50 (31,753.94) | 36,502.87 (17,759.93) | 40,023.77 (18,425.64) | 43,564.77 (18,873.47) | 46,682.06 (20,159.88) | 49,165.53 (21,579.54) | 53,015.81 (23,482.90) | 58,171.84 (25,830.74) | 62,800.19 (27,722.07) | 68,643.90 (31,783.95) | 70,725.58 (31,328.54) | 79,871.23 (35,193.79) | 84,284.68 (36,092.35) | 88,205.23 (38,001.87) | 4424.27 | < 0.001 |
| **Average years of education per capita (years)** | 9.22 (1.12) | 8.81 (1.01) | 8.89 (1.11) | 8.98 (1.19) | 8.96 (1.19) | 9.07 (1.11) | 9.10 (1.18) | 9.27 (1.15) | 9.27 (1.12) | 9.31 (1.12) | 9.14 (0.98) | 9.69 (1.03) | 9.70 (1.00) | 9.69 (1.03) | 0.07 | < 0.001 |

Notes: Values are presented as mean (standard deviation) across provincial-level regions in China. Fixed-effects panel regression models were used to assess the yearly trend from 2011 to 2023, with coefficient representing the estimated average annual change, and P indicating the statistical significance of the trend. Abbreviations: BI, Baidu index; PBI, per capita Baidu index; GDP, gross domestic product.

# **Supplementary Table S2.** The definition of the spatial neighbours of each province-level region in China.

| **Province-level region** | **Number** | **Neighbors** |
| --- | --- | --- |
| Anhui | 6 | Henan, Hubei, Jiangsu, Jiangxi, Shandong, Zhejiang |
| Beijing | 2 | Hebei, Tianjin |
| Chongqing | 5 | Guizhou, Hubei, Hunan, Shaanxi, Sichuan |
| Fujian | 4 | Guangdong, Jiangxi, Zhejiang |
| Gansu | 6 | Inner Mongolia, Ningxia, Qinghai, Shaanxi, Sichuan, Xinjiang |
| Guangdong | 4 | Fujian, Guangxi, Hainan, Hunan, Jiangxi |
| Guangxi | 4 | Guangdong, Guizhou, Hunan, Yunnan |
| Guizhou | 5 | Chongqing, Guangxi, Hunan, Sichuan, Yunnan |
| Hainan | 1 | Guangdong |
| Hebei | 7 | Beijing, Henan, Inner Mongolia, Liaoning, Shandong, Shanxi, Tianjin |
| Heilongjiang | 2 | Inner Mongolia, Jilin |
| Henan | 6 | Anhui, Hebei, Hubei, Shaanxi, Shandong, Shanxi |
| Hubei | 6 | Anhui, Chongqing, Henan, Hunan, Jiangxi, Shaanxi |
| Hunan | 6 | Chongqing, Guangdong, Guangxi, Guizhou, Hubei, Jiangxi |
| Inner Mongolia | 8 | Gansu, Hebei, Heilongjiang, Jilin, Liaoning, Ningxia, Shaanxi, Shanxi |
| Jiangsu | 4 | Anhui, Shandong, Shanghai, Zhejiang |
| Jiangxi | 6 | Anhui, Fujian, Guangdong, Hubei, Hunan, Zhejiang |
| Jilin | 3 | Heilongjiang, Inner Mongolia, Liaoning |
| Liaoning | 3 | Hebei, Inner Mongolia, Jilin |
| Ningxia | 3 | Gansu, Inner Mongolia, Shaanxi |
| Qinghai | 4 | Gansu, Sichuan, Tibet, Xinjiang |
| Shaanxi | 8 | Chongqing, Gansu, Henan, Hubei, Inner Mongolia, Ningxia, Shanxi, Sichuan |
| Shandong | 4 | Anhui, Hebei, Henan, Jiangsu |
| Shanghai | 2 | Jiangsu, Zhejiang |
| Shanxi | 4 | Hebei, Henan, Inner Mongolia, Shaanxi |
| Sichuan | 7 | Chongqing, Gansu, Guizhou, Qinghai, Shaanxi, Tibet, Yunnan |
| Tianjin | 2 | Beijing, Hebei |
| Tibet | 4 | Qinghai, Sichuan, Xinjiang, Yunnan |
| Xinjiang | 3 | Gansu, Qinghai, Tibet |
| Yunnan | 4 | Guangxi, Guizhou, Sichuan, Tibet |
| Zhejiang | 5 | Anhui, Fujian, Jiangsu, Jiangxi, Shanghai |

# **Supplementary Table S3.** Results of variance inflation factor test for explanatory variables.

| **Variable** | **VIF** |
| --- | --- |
| Male-female ratio | 1.05 |
| Old-age dependency ratio | 1.33 |
| Urbanization rate | 6.85 |
| GDP per capita | 3.2 |
| Average years of education per capita | 4.32 |

Abbreviations: VIF, variance inflation factor; GDP, gross domestic product.

# **Supplementary Table S4.** Results of the spatial panel econometric model selection test.

| **Spatial panel model test** | | **Alternative hypothesis** | **Statistic** | *P* **value** |
| --- | --- | --- | --- | --- |
| LM test | LM-error | Spatial error dependence | 325.2 | < 0.001 |
|  | LM-lag | Spatial lag dependence | 319.39 | < 0.001 |
|  | Robust-LM-error | Spatial error dependence | 10.2 | 0.001 |
|  | Robust-LM-lag | Spatial lag dependence | 4.39 | 0.036 |
| Hausman test | / | The random effects estimator is inconsistent | 13.57 | 0.02 |
| Wald test | Wald-SDM/SAR | The SDM model cannot be simplified to SAR model | 102.17 | < 0.001 |
|  | Wald-SDM/SEM | The SDM model cannot be simplified to SEM model | 30.02 | < 0.001 |
| LR test | LR-both/space | Time fixed effects are significant | 121.68 | < 0.001 |
|  | LR-both/time | Spatial fixed effects are significant | 905.31 | < 0.001 |

Abbreviations: LM test, Lagrange multiplier test; SDM, spatial Durbin model; SAR, spatial autoregressive model; SEM, spatial error model; likelihood ratio.

# **Supplementary Table S5.** Emotion Taxonomy and Example Lexemes of DLUT-Emotion Ontology.

| **Category** | **Example lexemes** |
| --- | --- |
| Happy (乐) | joyful (喜悦), delighted (欢喜), reassured (踏实), relieved (宽心) |
| Good (好) | respectful (恭敬), devoted (敬爱), admirable (英俊), excellent (优秀), trust (信任), rely on (信赖), reliable (可靠), adore (倾慕) |
| Anger (怒) | angry (气愤), annoyed (恼火), furious (大发雷霆) |
| Sad (哀) | sorrowful (忧伤), wretched (悲苦), dejected (懊丧), despairing (绝望), guilty (内疚), repentant (忏悔), yearning (相思), miss (思念) |
| Fear (惧) | flustered (慌张), panicky (心慌), timid (胆怯), afraid (害怕), shy (害羞), ashamed (羞愧) |
| Disgust (恶) | troubled (烦闷), irritable (烦躁), averse (反感), stiff/rigid (呆板), vain (虚荣), envious (眼红), jealous (吃醋), suspicious (生疑) |
| Surprise (惊) | strange/odd (奇怪), wondrous (奇迹), astonished (大吃一惊) |

Notes: Terms are organized into seven categories. English glosses are approximate; Chinese originals are retained in parentheses for precision. Abbreviations: DLUT, Dalian University of Technology emotion ontology.

# **Supplementary Table S6.** The concentration ratio of the daily average Baidu index for lung cancer, 2011-2023.

| **Year** | **January** | **February** | **March** | **April** | **May** | **June** | **July** | **August** | **September** | **October** | **November** | **December** | **M** |
| --- | --- | --- | --- | --- | --- | --- | --- | --- | --- | --- | --- | --- | --- |
| 2011 | 2238.516 | 2443.929 | 2649.839 | 2735.3 | 2769.129 | 2658.767 | 2707.387 | 2632.581 | 2648.767 | 2709.839 | 3160.2 | 3530.935 | 0.03 |
| 2012 | 3357.226 | 4177.828 | 4300.581 | 4297.1 | 4242.903 | 3779.267 | 3529.323 | 3716.387 | 3362.467 | 3411.806 | 3293.3 | 3023.645 | 0.07 |
| 2013 | 3481.355 | 3364.679 | 3689.613 | 3408.333 | 3526.677 | 3596.3 | 3897.968 | 3886.194 | 4020.5 | 3934.613 | 4011.167 | 4033.903 | 0.04 |
| 2014 | 3928.323 | 4285.857 | 4045.71 | 4148.167 | 4079.645 | 3924.867 | 3982.194 | 4074.194 | 4217.233 | 4393.161 | 4490.3 | 4505.645 | 0.02 |
| 2015 | 4847.226 | 4485.036 | 4834.484 | 4687.233 | 4422.355 | 4454.067 | 4534.29 | 4475.581 | 4210.567 | 4140.258 | 4233.667 | 4556.742 | 0.03 |
| 2016 | 4364.161 | 4197.414 | 4900.774 | 5133.233 | 6494.516 | 4527.067 | 3965.742 | 4038.871 | 4158.7 | 4094.645 | 4267.633 | 4627.742 | 0.07 |
| 2017 | 4331.806 | 4434.5 | 5621.871 | 5943.967 | 6401.387 | 6243.567 | 6184.355 | 6410.032 | 6123.067 | 4544.419 | 4364.067 | 4101.581 | 0.11 |
| 2018 | 4694.71 | 4151 | 4905.129 | 4695.867 | 5596.774 | 6443.567 | 7384.29 | 7260.839 | 6626.233 | 6796.935 | 7028.367 | 7560.29 | 0.11 |
| 2019 | 7524.774 | 7070.071 | 8796.323 | 9042.633 | 9119.355 | 8465.8 | 6818.871 | 6334.516 | 5635.333 | 5630.548 | 5507.733 | 6590.032 | 0.12 |
| 2020 | 5648.226 | 5108.172 | 5695.613 | 5505.067 | 5101.194 | 5068.3 | 5926.581 | 5376.548 | 5297.367 | 4944.677 | 5370.667 | 5103.71 | 0.01 |
| 2021 | 4983.516 | 5069.821 | 5986.742 | 5985.867 | 5594.677 | 5775.067 | 5680.161 | 5683.968 | 6192.267 | 5483.387 | 5861.867 | 6111.806 | 0.01 |
| 2022 | 5740.935 | 5662.679 | 5689.548 | 5370.533 | 5473.323 | 5441.033 | 5497.581 | 5372.097 | 4928.267 | 4657.968 | 4655.033 | 3982.161 | 0.05 |
| 2023 | 4081.323 | 4790.714 | 4931.065 | 4661.4 | 4276.032 | 4140.933 | 4067.935 | 4111.645 | 3918.7 | 3682.161 | 3751.167 | 3650.29 | 0.06 |
| 2024 | 3759.968 | 3561.621 | 3994.226 | 3735.967 | 3600.968 | 3502.233 | 3670.323 | 4614.323 | 3686.6 | 3950.677 | 3797.633 | 3602.548 | 0.02 |
| 2025 | 3604.194 | 3457.929 | 3449.516 | 3457.667 | 3240.806 | 3213.267 | 2890.548 | 2807.419 | NA | NA | NA | NA | - |

# **Supplementary Table S7.** Fifty lung cancer–related topics on Weibo with top 20 keywords and manually annotated themes.

| **Topic No.** | **Top Words** | **No. of Posts** | **Theme** |
| --- | --- | --- | --- |
| 1 | Genetic testing, gene, testing, institution, service, heredity, consultation, provide, disease, appointment, patient, professional, sample, sampling, selection, tumor, Zhongtian, Zhongyun, Zhongbo, center | 4735 | Healthcare service |
| 2 | Treatment, patient, combination, non-small cell lung cancer, monoclonal antibody, used for, first-line, chemotherapy, approval, clinical trial, market launch, drug, PD-1, approval granted, indication, clinical, inhibitor, research, NSCLC, FDA | 9245 | Treatment |
| 3 | Health, exercise, lifestyle, body, workout, sleep, mode, male, prevention, reduce, decrease, hours, improve, immunity, disease, risk, knowledge, beneficial, diet, stress | 4368 | Prevention |
| 4 | Patient, treatment, traditional Chinese medicine, Chinese herbal medicine, anti-cancer, effect, condition, disappearance, advanced stage, patient, lung cancer, take (medication), Western medicine, recuperation, medicine, relief, method, family member, improvement, recovery | 5976 | Traditional Chinese medicine |
| 5 | Smoking, smoking cessation, secondhand smoke, tobacco, harm, health, cause, non-smoker, disease, risk, smoke, exposure, increase, cigarette, smoker, multiple, nicotine, impact, passive, harmful | 12350 | Prevention |
| 6 | Advanced stage, diagnosis, lung adenocarcinoma, lung cancer, video, one year, detected, surgery, hope, wife, last year, family, anti-cancer, Shanghai, netizen, friend, aunt, one person, life, lady | 5987 | Personal experience |
| 7 | Hospital, hospitalization, elderly, evening, feeling, several days, go home, colleague, afternoon, really, yesterday, morning, only, a little, family member, not well, ward, tomorrow, tell, father | 11147 | Personal experience |
| 8 | Disease, diabetes, health, hypertension, cardiovascular, not suitable, heart disease, elderly, blood pressure, prevention, children, blood glucose, cardio-cerebrovascular, blood vessel, decrease, weekly, skin, increase, osteoporosis, impact | 2513 | Comorbidities |
| 9 | Infection, virus, COVID-19, pneumonia, patient, vaccine, disease, severe case, epidemic, case, vaccination, underlying condition, fever, situation, symptom, infected person, asymptomatic, population, hospital, treatment | 3555 | Comorbidities |
| 10 | Patient, treatment, chemotherapy, radiotherapy, tumor, regimen, drug, effect, survival, recurrence, immunotherapy, choice, quality, method, condition, prolongation, situation, chemoradiotherapy, side effect, advanced stage | 6360 | Treatment |
| 11 | Lung cancer, smoking, video, developed, detected, diagnosis, Weibo, family, man, advanced stage, drinking, woman, recently, Henan, long-term, couple, husband, discovery, doctor, Zhengzhou | 9728 | Personal experience |
| 12 | Cooking oil fume, pollution, air, indoor, environment, smog, kitchen, renovation, PM, kitchen fumes, air pollution, impact, reduce, human body, inhalation, harm, ventilation, gas, cause, formaldehyde | 7265 | Prevention |
| 13 | Death, lung cancer, teacher, elderly, Beijing, news, one person, aged, passing away, advanced stage, student, donation, one individual, remains, actor, deceased, family, netizen, all the way, renowned | 7590 | Personal experience |
| 14 | Treatment, patient, mutation, EGFR, targeted drug, non-small cell lung cancer, advanced stage, drug resistance, ALK, positive, drug, gene mutation, NSCLC, tinib, inhibitor, targeted therapy, gene, KRAS, TKI, progression | 7234 | Treatment |
| 15 | Drug, national, medical insurance, medication, cost, average, price, procurement, disease, inclusion, patient, including, organization, treatment, selected, price reduction, negotiation, healthcare, centralized procurement, burden | 2981 | Healthcare service |
| 16 | Examination, screening, physical examination, CT, breast, recommendation, testing, age 40, detection, elevated, item, tumor marker, ultrasound, age 50, dosage, method, high-risk population, diagnosis, indicator, B-ultrasound | 4997 | Screening and diagnosis |
| 17 | Body, emotion, influence, pressure, state, mental, psychology, anxiety, cause, increasingly, life, disease, work, long-term, mood, energy, anger, health, depression, excessive | 2678 | Prevention |
| 18 | Food, risk, increase, diet, cause, food products, long-term, intake, occurrence, fat, obesity, recommendation, nutrition, habit, intestine, alcohol consumption, carcinogenic, digestion, carcinogen, induce | 4337 | Prevention |
| 19 | China, United States, world, Japan, human, country, international, report, Hong Kong, one type, lung cancer, science, life expectancy, time, society, death, plan, history, indicate, electronic | 3015 | Health communication |
| 20 | Cell, immunity, tumor, cancer cell, function, inhibition, system, function, human body, in vivo, growth, one type, tissue, occurrence, organism, ability, mechanism, gene, organ, cause | 3451 | Academic research |
| 21 | Lung cancer, early stage, detection, screening, physical examination, advanced stage, diagnosis, cure, symptom, survival rate, reminder, emphasize, high-risk population, key, improvement, chest radiograph, large, dosage, health, prevention and treatment | 6501 | Screening and diagnosis |
| 22 | Treatment, patient, cell, lung cancer, therapy, tumor, novel, hope, one type, bring, small cell lung cancer, institution, immunity, tumor mass, CT, traditional, method, effect, immunotherapy, choice | 1819 | Treatment |
| 23 | Father, mother, hope, family, love, gratitude, family member, friend, home, help request, kind-hearted person, individual, hospital, thanks, cost, society, link, life, this is, diagnosis | 6749 | Personal Experience |
| 24 | Lung, treatment, phlegm, traditional Chinese medicine, licorice, efficacy, effect, pinellia, resolve phlegm, used for, clinical, astragalus, prescription, poria, dissipate nodule, clear heat and detoxify, detoxify, ginseng, cough, phlegm-dampness | 5558 | Traditional Chinese medicine |
| 25 | Symptom, pain, manifestation, signal, body, vigilance, cause, situation, abnormal, nerve, reason, lump, examination, site, persistent, skin, hoarseness, compression, ache, joint | 6489 | Symptoms |
| 26 | Lung, human body, traditional Chinese medicine, body, kidney, heart, function, heat, qi, qi and blood, constitution, lung, poor, liver, method, in vivo, health preservation, moisten lung, large intestine, common cold | 3578 | Traditional Chinese medicine |
| 27 | Pulmonary nodule, nodule, malignant, lung, benign, CT, ground-glass nodule, follow-up, lung, detection, lesion, pulmonary nodule, size, millimeter, diameter, thyroid, follow-up, change, density, small nodule | 9050 | Screening and diagnosis |
| 28 | Healthcare, technology, diagnosis, medicine, clinical, development, precision, provide, team, improvement, AI, medical treatment, field, health, system, imaging, disease, information, level, capacity | 3633 | Healthcare service |
| 29 | Healthcare, insurance, physical examination, company, lady, paternity test, insurance company, claim, court, diagnosis, notification, situation, client, hospital, responsibility, final, insurance, ten thousand yuan, appraisal, compensation | 2916 | Healthcare service |
| 30 | Child, son, daughter, parent, husband, spouse, care, two, wife, marriage, home, mother-in-law, man, house, mother, wife, work, woman, elderly, death | 5591 | Personal experience |
| 31 | Mother, father, grandfather, really, grandmother, death, maternal grandmother, grandfather (maternal), hope, leave, take good care, maternal grandfather, go home, family, sad, remember, especially, my mother, maternal grandmother, seems | 12968 | Personal experience |
| 32 | Female, incidence, cancer, death, our country, mortality rate, male, global, case, incidence, China, data, malignant, show, breast cancer, first, number, tumor, new case, country | 10252 | Health communication |
| 33 | Surgery, patient, postoperative, treatment, resection, recovery, lung, puncture, lung, thoracic surgery, success, doctor, pathology, lesion, diagnosis, tissue, tumor, intervention, biopsy, thoracic surgery | 4698 | Treatment |
| 34 | Someone, really, indeed, friend, a bit, certainly, special, thing, two, because, tell, time, whether, reason, simple, this is, one type, only, worry, understanding | 3393 | Personal experience |
| 35 | Hospital, expert, tumor, professor, director, affiliated, center, medicine, cancer hospital, internal medicine, live broadcast, respiratory, thoracic surgery, chief physician, people’s hospital, Beijing, medical treatment, China, oncology, professional | 8012 | Healthcare service |
| 36 | Time, minute, adjustment, temperature, fermentation, extraction, stirring, exhaust port, stage, cell, air inlet, diameter, speed, crushing, antibody, mm, first time, packaging, state, open | 191 | Academic research |
| 37 | Life, living, life course, story, world, face, experience, effort, pain, lifetime, forever, movie, feeling, death, Guilin, hope, choice, beautiful, destiny, matter | 7855 | Personal experience |
| 38 | Cancer, breast cancer, liver cancer, gastric cancer, colorectal cancer, esophageal cancer, cancer, cervical cancer, prostate cancer, pancreatic cancer, prevention, thyroid cancer, intestinal cancer, colon cancer, ovarian cancer, leukemia, kidney cancer, cancer prevention, bladder cancer, common | 4313 | Academic research |
| 39 | Doctor, hospital, examination, recommendation, detection, lung, Weibo, free, situation, viewing, answer, medical visit, lady, consultation, value, reminder, condition, CT, diagnosis, Q&A | 5112 | Health communication |
| 40 | Lung cancer, advanced stage, expert, click, science popularization, details, life, incidence, colorectal cancer, November, introduction, younger trend, reason, sedentary, trend, 70 years, keep away, neglect, steward, Times | 2815 | Health communication |
| 41 | Prevention, vitamin, reduce, effect, contain, anti-cancer, inhibit, nutrition, rich in, apple, consumption, substance, fiber, vegetable, component, food, function, garlic, supplement, human body | 7087 | Prevention |
| 42 | Research, discovery, risk, related, one study, cancer, personnel, analysis, increase, decrease, show, United States, impact, compared with, one type, scientist, this study, journal, among, data | 3920 | Academic research |
| 43 | Cell, expression, specificity, affinity, drug, tumor, protein, process, treatment, antibody, direction, CL, related, strain, product, simple, immunity, conjugation, fusion, antigen | 567 | Academic research |
| 44 | Disease, blood vessel, blood, heart, cause, bleeding, acute, chronic, function, treatment, liver, thrombosis, skin, symptom, occurrence, patient, vein, hormone, syndrome, system | 2588 | Comorbidities |
| 45 | Company, innovation, biology, product, research and development, pharmaceutical, China, drug, market, global, field, enterprise, clinical, market launch, billion yuan, domestic, growth, pharmaceutical industry, billion USD, industry | 3153 | Healthcare service |
| 46 | Looking, eye, really, voice, friend, mobile phone, reading, full text, aunt, tell, man, one photo, place, like, seems, photo, one bite, protagonist, hospital, sitting | 4470 | Personal experience |
| 47 | Tumor, metastasis, malignant, cancer, occurrence, tissue, recurrence, site, type, adenocarcinoma, pathology, lymph node, lymphoma, common, neoplasm, spread, prognosis, degree, growth, multiple | 3253 | Treatment |
| 48 | Work, project, health, hygiene, community, activity, service, hospital, resident, free, reporter, participation, situation, publicity, mass, personnel, related, city, survey, unit | 3472 | Healthcare service |
| 49 | Factor, lung cancer, population, occurrence, smoking, risk, long-term, heredity, environment, prevention, age, cause, reason, disease, exposure, lifestyle, family history, include, high-risk, risk factor | 4011 | Prevention |
| 50 | Cough, lung, pulmonary, symptom, disease, respiration, chronic, phlegm, pneumonia, bronchus, infection, cause, tuberculosis, patient, respiratory tract, chest tightness, chest pain, manifestation, hemoptysis, asthma | 5230 | Symptoms |

# **Supplementary Table S8.** Sentiment analysis results for Weibo posts on lung cancer by subgroup.

| **Group** | | **Total score** | | **Happy** | | **Good** | | **Anger** | | **Sad** | | **Fear** | | **Disgust** | | **Surprise** |
| --- | --- | --- | --- | --- | --- | --- | --- | --- | --- | --- | --- | --- | --- | --- | --- | --- |
| **Mean (SD)** | |  | |  | |  | |  | |  | |  | |  | |  |
| **All posts** | | 8.56 (28.41) | | 3.04 (8.11) | | 14.60 (27.43) | | -0.16 (1.82) | | -0.69 (2.99) | | -1.40 (6.04) | | -7.01 (14.80) | | 0.16 (1.33) |
| **Year** | |  | |  | |  | |  | |  | |  | |  | |  |
| 2010 | | 1.44 (5.45) | | 0.28 (1.15) | | 2.15 (4.89) | | 0.00 (0.00) | | -0.01 (1.28) | | -0.10 (0.82) | | -0.88 (2.39) | | 0.00 (0.00) |
| 2011 | | 1.62 (6.55) | | 0.34 (1.50) | | 3.07 (5.28) | | 0.00 (0.00) | | -0.27 (1.26) | | -0.18 (1.05) | | -1.47 (2.93) | | 0.14 (1.26) |
| 2012 | | 4.40 (8.73) | | 0.64 (2.18) | | 5.78 (6.95) | | -0.02 (0.34) | | -0.24 (1.35) | | -0.32 (1.57) | | -1.49 (3.53) | | 0.04 (0.54) |
| 2013 | | 1.84 (7.43) | | 0.37 (1.67) | | 4.61 (5.69) | | -0.01 (0.26) | | -0.16 (1.11) | | -0.20 (1.18) | | -2.78 (5.31) | | 0.01 (0.18) |
| 2014 | | 4.44 (8.02) | | 0.61 (2.65) | | 6.31 (5.89) | | -0.00 (0.13) | | -0.28 (1.45) | | -0.93 (3.14) | | -1.29 (3.09) | | 0.02 (0.64) |
| 2015 | | 3.36 (8.00) | | 0.79 (2.46) | | 4.18 (5.96) | | 0.00 (0.00) | | -0.17 (1.26) | | -0.21 (1.16) | | -1.26 (3.56) | | 0.04 (0.95) |
| 2016 | | 7.07 (17.85) | | 1.98 (5.36) | | 10.37 (15.43) | | -0.06 (0.87) | | -0.37 (1.81) | | -0.98 (3.84) | | -4.11 (9.10) | | 0.24 (1.91) |
| 2017 | | 7.27 (20.64) | | 2.34 (5.96) | | 12.36 (19.16) | | -0.12 (1.39) | | -0.42 (2.63) | | -1.24 (4.29) | | -5.84 (11.96) | | 0.18 (1.33) |
| 2018 | | 8.30 (23.84) | | 2.64 (6.54) | | 13.85 (23.63) | | -0.12 (1.44) | | -0.65 (2.68) | | -1.35 (4.84) | | -6.26 (13.07) | | 0.18 (1.40) |
| 2019 | | 6.01 (20.92) | | 2.06 (5.48) | | 10.45 (19.56) | | -0.10 (1.55) | | -0.46 (2.34) | | -0.93 (4.30) | | -5.17 (11.30) | | 0.15 (1.29) |
| 2020 | | 6.68 (23.94) | | 2.59 (6.69) | | 12.66 (22.24) | | -0.15 (1.61) | | -0.70 (3.11) | | -1.55 (7.18) | | -6.35 (12.79) | | 0.18 (1.44) |
| 2021 | | 6.49 (22.76) | | 2.54 (6.87) | | 11.70 (20.93) | | -0.13 (1.87) | | -0.67 (2.88) | | -1.37 (5.63) | | -5.72 (11.85) | | 0.15 (1.29) |
| 2022 | | 6.92 (28.14) | | 2.88 (7.90) | | 13.30 (26.84) | | -0.16 (1.77) | | -0.76 (3.14) | | -1.77 (9.28) | | -6.74 (14.73) | | 0.15 (1.24) |
| 2023 | | 8.30 (30.20) | | 2.84 (8.32) | | 15.01 (30.54) | | -0.15 (1.70) | | -0.78 (3.18) | | -1.39 (4.87) | | -7.41 (16.25) | | 0.17 (1.31) |
| 2024 | | 10.36 (33.17) | | 3.38 (9.01) | | 17.40 (32.96) | | -0.17 (1.71) | | -0.69 (3.17) | | -1.47 (5.59) | | -8.24 (16.60) | | 0.15 (1.30) |
| 2025 | | 17.24 (40.62) | | 6.34 (12.51) | | 24.93 (37.07) | | -0.33 (2.94) | | -0.91 (3.39) | | -1.43 (5.36) | | -11.54 (20.21) | | 0.18 (1.41) |
| **Gender/Organization** | |  | |  | |  | |  | |  | |  | |  | |  |
| Male | | 8.30 (29.09) | | 2.69 (7.88) | | 15.40 (29.80) | | -0.15 (1.62) | | -0.76 (3.32) | | -1.46 (5.67) | | -7.60 (16.30) | | 0.18 (1.40) |
| Female | | 5.77 (22.06) | | 2.50 (6.51) | | 11.63 (22.33) | | -0.13 (1.50) | | -0.80 (3.00) | | -0.91 (4.15) | | -6.72 (14.01) | | 0.20 (1.51) |
| Organization | | 11.20 (32.09) | | 3.87 (9.42) | | 16.34 (28.65) | | -0.19 (2.21) | | -0.52 (2.58) | | -1.76 (7.56) | | -6.64 (13.81) | | 0.11 (1.05) |
| **User type** | |  | |  | |  | |  | |  | |  | |  | |  |
| Government | | 8.57 (29.59) | | 2.92 (7.24) | | 13.87 (26.68) | | -0.14 (2.04) | | -0.49 (2.23) | | -1.83 (7.01) | | -5.87 (11.81) | | 0.10 (0.92) |
| Health professionals | | 9.02 (27.58) | | 3.05 (7.81) | | 16.52 (27.01) | | -0.18 (2.09) | | -0.66 (2.71) | | -1.47 (5.35) | | -8.37 (16.40) | | 0.13 (1.18) |
| Media outlets | | 10.81 (34.63) | | 3.82 (10.36) | | 15.84 (30.03) | | -0.17 (2.03) | | -0.57 (2.87) | | -2.05 (9.65) | | -6.20 (12.80) | | 0.14 (1.10) |
| General public | | 7.96 (26.98) | | 2.90 (7.79) | | 13.92 (27.07) | | -0.15 (1.64) | | -0.76 (3.18) | | -1.18 (5.00) | | -6.97 (15.10) | | 0.19 (1.45) |
| **Frame** | |  | |  | |  | |  | |  | |  | |  | |  |
| Prevention | | 4.94 (24.37) | | 1.86 (5.60) | | 14.20 (25.28) | | -0.08 (0.92) | | -0.80 (2.63) | | -2.01 (5.82) | | -8.27 (16.04) | | 0.06 (0.69) |
| Symptoms | | -2.74 (17.60) | | 1.50 (4.54) | | 9.01 (14.04) | | -0.06 (0.74) | | -0.51 (2.09) | | -1.63 (6.15) | | -11.16 (16.20) | | 0.10 (0.93) |
| Screening and diagnosis | | 4.42 (16.16) | | 1.44 (5.73) | | 10.47 (15.41) | | -0.04 (0.76) | | -0.28 (1.71) | | -0.74 (3.69) | | -6.51 (12.00) | | 0.09 (0.78) |
| Treatment | | 8.84 (20.20) | | 3.41 (7.43) | | 10.65 (19.01) | | -0.14 (1.63) | | -0.37 (1.82) | | -0.77 (3.89) | | -4.10 (9.86) | | 0.14 (1.19) |
| TCM | | 9.22 (28.30) | | 3.11 (7.86) | | 19.95 (34.60) | | -0.26 (1.79) | | -1.52 (5.14) | | -1.50 (4.58) | | -10.82 (20.27) | | 0.27 (1.77) |
| Comorbidities | | -3.78 (30.44) | | 2.24 (7.24) | | 14.51 (26.99) | | -0.19 (2.85) | | -0.77 (2.69) | | -7.38 (20.88) | | -12.27 (22.46) | | 0.09 (1.27) |
| Personal experience | | 8.17 (24.26) | | 3.01 (7.03) | | 13.42 (25.03) | | -0.12 (1.00) | | -0.99 (3.74) | | -0.64 (2.61) | | -6.82 (13.97) | | 0.31 (1.89) |
| Healthcare service | | 28.22 (49.36) | | 8.09 (15.08) | | 28.95 (43.90) | | -0.48 (3.85) | | -0.45 (2.67) | | -0.79 (3.62) | | -7.18 (16.81) | | 0.08 (0.92) |
| Health communication | | 4.08 (15.82) | | 1.42 (4.11) | | 7.93 (15.14) | | -0.08 (1.16) | | -0.22 (1.77) | | -1.98 (5.75) | | -3.06 (8.13) | | 0.08 (0.89) |
| Academic research | | 9.55 (33.64) | | 1.89 (7.19) | | 17.17 (35.12) | | -0.18 (2.70) | | -0.46 (2.24) | | -2.58 (9.21) | | -6.36 (14.52) | | 0.07 (0.85) |
| **Median (Q1, Q3)** | |  | |  | |  | |  | |  | |  | |  | |  |
| **All posts** | 3.00 (-1.00, 12.00) | | 0.00 (0.00, 3.00) | | 6.00 (0.00, 16.00) | | 0.00 (0.00, 0.00) | | 0.00 (0.00, 0.00) | | 0.00 (0.00, 0.00) | | 0.00 (-8.00, 0.00) | | 0.00 (0.00, 0.00) | |
| **Year** |  | |  | |  | |  | |  | |  | |  | |  | |
| 2010 | 0.00 (0.00, 3.00) | | 0.00 (0.00, 0.00) | | 0.00 (0.00, 3.00) | | 0.00 (0.00, 0.00) | | 0.00 (0.00, 0.00) | | 0.00 (0.00, 0.00) | | 0.00 (0.00, 0.00) | | 0.00 (0.00, 0.00) | |
| 2011 | 0.00 (0.00, 5.00) | | 0.00 (0.00, 0.00) | | 0.00 (0.00, 5.00) | | 0.00 (0.00, 0.00) | | 0.00 (0.00, 0.00) | | 0.00 (0.00, 0.00) | | 0.00 (0.00, 0.00) | | 0.00 (0.00, 0.00) | |
| 2012 | 3.00 (0.00, 10.00) | | 0.00 (0.00, 0.00) | | 3.00 (0.00, 10.00) | | 0.00 (0.00, 0.00) | | 0.00 (0.00, 0.00) | | 0.00 (0.00, 0.00) | | 0.00 (0.00, 0.00) | | 0.00 (0.00, 0.00) | |
| 2013 | 0.00 (0.00, 5.00) | | 0.00 (0.00, 0.00) | | 3.00 (0.00, 8.00) | | 0.00 (0.00, 0.00) | | 0.00 (0.00, 0.00) | | 0.00 (0.00, 0.00) | | 0.00 (-3.00, 0.00) | | 0.00 (0.00, 0.00) | |
| 2014 | 5.00 (0.00, 9.00) | | 0.00 (0.00, 0.00) | | 6.00 (0.00, 9.00) | | 0.00 (0.00, 0.00) | | 0.00 (0.00, 0.00) | | 0.00 (0.00, 0.00) | | 0.00 (0.00, 0.00) | | 0.00 (0.00, 0.00) | |
| 2015 | 1.00 (0.00, 7.00) | | 0.00 (0.00, 0.00) | | 0.50 (0.00, 6.00) | | 0.00 (0.00, 0.00) | | 0.00 (0.00, 0.00) | | 0.00 (0.00, 0.00) | | 0.00 (0.00, 0.00) | | 0.00 (0.00, 0.00) | |
| 2016 | 4.00 (0.00, 13.00) | | 0.00 (0.00, 3.00) | | 6.00 (0.00, 14.00) | | 0.00 (0.00, 0.00) | | 0.00 (0.00, 0.00) | | 0.00 (0.00, 0.00) | | 0.00 (-5.00, 0.00) | | 0.00 (0.00, 0.00) | |
| 2017 | 5.00 (0.00, 13.00) | | 0.00 (0.00, 3.00) | | 6.00 (0.00, 15.00) | | 0.00 (0.00, 0.00) | | 0.00 (0.00, 0.00) | | 0.00 (0.00, 0.00) | | 0.00 (-6.00, 0.00) | | 0.00 (0.00, 0.00) | |
| 2018 | 3.00 (0.00, 13.00) | | 0.00 (0.00, 3.00) | | 6.00 (0.00, 16.00) | | 0.00 (0.00, 0.00) | | 0.00 (0.00, 0.00) | | 0.00 (0.00, 0.00) | | 0.00 (-7.00, 0.00) | | 0.00 (0.00, 0.00) | |
| 2019 | 3.00 (0.00, 10.00) | | 0.00 (0.00, 3.00) | | 5.00 (0.00, 12.00) | | 0.00 (0.00, 0.00) | | 0.00 (0.00, 0.00) | | 0.00 (0.00, 0.00) | | 0.00 (-5.00, 0.00) | | 0.00 (0.00, 0.00) | |
| 2020 | 3.00 (0.00, 11.00) | | 0.00 (0.00, 3.00) | | 6.00 (0.00, 15.00) | | 0.00 (0.00, 0.00) | | 0.00 (0.00, 0.00) | | 0.00 (0.00, 0.00) | | 0.00 (-8.00, 0.00) | | 0.00 (0.00, 0.00) | |
| 2021 | 2.00 (-1.00, 11.00) | | 0.00 (0.00, 3.00) | | 5.00 (0.00, 14.00) | | 0.00 (0.00, 0.00) | | 0.00 (0.00, 0.00) | | 0.00 (0.00, 0.00) | | 0.00 (-7.00, 0.00) | | 0.00 (0.00, 0.00) | |
| 2022 | 2.00 (-2.00, 11.00) | | 0.00 (0.00, 3.00) | | 5.00 (0.00, 14.00) | | 0.00 (0.00, 0.00) | | 0.00 (0.00, 0.00) | | 0.00 (0.00, 0.00) | | 0.00 (-8.00, 0.00) | | 0.00 (0.00, 0.00) | |
| 2023 | 2.00 (-2.00, 12.00) | | 0.00 (0.00, 3.00) | | 5.00 (0.00, 16.00) | | 0.00 (0.00, 0.00) | | 0.00 (0.00, 0.00) | | 0.00 (0.00, 0.00) | | 0.00 (-8.00, 0.00) | | 0.00 (0.00, 0.00) | |
| 2024 | 3.00 (0.00, 13.00) | | 0.00 (0.00, 3.00) | | 7.00 (0.00, 18.00) | | 0.00 (0.00, 0.00) | | 0.00 (0.00, 0.00) | | 0.00 (0.00, 0.00) | | -3.00 (-10.00, 0.00) | | 0.00 (0.00, 0.00) | |
| 2025 | 5.00 (0.00, 24.00) | | 0.00 (0.00, 9.00) | | 10.00 (3.00, 34.00) | | 0.00 (0.00, 0.00) | | 0.00 (0.00, 0.00) | | 0.00 (0.00, 0.00) | | -5.00 (-15.00, 0.00) | | 0.00 (0.00, 0.00) | |
| **Gender/Organization** |  | |  | |  | |  | |  | |  | |  | |  | |
| Male | 3.00 (0.00, 12.00) | | 0.00 (0.00, 3.00) | | 6.00 (0.00, 17.00) | | 0.00 (0.00, 0.00) | | 0.00 (0.00, 0.00) | | 0.00 (0.00, 0.00) | | 0.00 (-9.00, 0.00) | | 0.00 (0.00, 0.00) | |
| Female | 0.00 (-1.00, 10.00) | | 0.00 (0.00, 3.00) | | 5.00 (0.00, 14.00) | | 0.00 (0.00, 0.00) | | 0.00 (0.00, 0.00) | | 0.00 (0.00, 0.00) | | 0.00 (-8.00, 0.00) | | 0.00 (0.00, 0.00) | |
| Organization | 5.00 (0.00, 15.00) | | 0.00 (0.00, 5.00) | | 7.00 (3.00, 17.00) | | 0.00 (0.00, 0.00) | | 0.00 (0.00, 0.00) | | 0.00 (0.00, 0.00) | | 0.00 (-8.00, 0.00) | | 0.00 (0.00, 0.00) | |
| **User type** |  | |  | |  | |  | |  | |  | |  | |  | |
| Government | 3.00 (-2.00, 12.00) | | 0.00 (0.00, 3.00) | | 6.00 (3.00, 14.00) | | 0.00 (0.00, 0.00) | | 0.00 (0.00, 0.00) | | 0.00 (0.00, 0.00) | | -1.00 (-6.00, 0.00) | | 0.00 (0.00, 0.00) | |
| Health professionals | 3.00 (0.00, 13.00) | | 0.00 (0.00, 3.00) | | 8.00 (0.00, 19.00) | | 0.00 (0.00, 0.00) | | 0.00 (0.00, 0.00) | | 0.00 (0.00, 0.00) | | -1.00 (-10.00, 0.00) | | 0.00 (0.00, 0.00) | |
| Media outlets | 5.00 (-1.00, 15.00) | | 0.00 (0.00, 4.00) | | 8.00 (3.00, 17.00) | | 0.00 (0.00, 0.00) | | 0.00 (0.00, 0.00) | | 0.00 (0.00, 0.00) | | 0.00 (-7.00, 0.00) | | 0.00 (0.00, 0.00) | |
| General public | 2.00 (0.00, 12.00) | | 0.00 (0.00, 3.00) | | 5.00 (0.00, 15.00) | | 0.00 (0.00, 0.00) | | 0.00 (0.00, 0.00) | | 0.00 (0.00, 0.00) | | 0.00 (-8.00, 0.00) | | 0.00 (0.00, 0.00) | |
| **Frame** |  | |  | |  | |  | |  | |  | |  | |  | |
| Prevention | 1.00 (-3.00, 10.00) | | 0.00 (0.00, 1.00) | | 6.00 (0.00, 15.00) | | 0.00 (0.00, 0.00) | | 0.00 (0.00, 0.00) | | 0.00 (0.00, 0.00) | | -3.00 (-10.00, 0.00) | | 0.00 (0.00, 0.00) | |
| Symptoms | 0.00 (-8.00, 4.00) | | 0.00 (0.00, 3.00) | | 5.00 (0.00, 12.00) | | 0.00 (0.00, 0.00) | | 0.00 (0.00, 0.00) | | 0.00 (0.00, 0.00) | | -5.00 (-15.00, 0.00) | | 0.00 (0.00, 0.00) | |
| Screening and diagnosis | 3.00 (0.00, 10.00) | | 0.00 (0.00, 3.00) | | 6.00 (0.00, 13.00) | | 0.00 (0.00, 0.00) | | 0.00 (0.00, 0.00) | | 0.00 (0.00, 0.00) | | -1.00 (-8.00, 0.00) | | 0.00 (0.00, 0.00) | |
| Treatment | 4.00 (0.00, 12.00) | | 0.00 (0.00, 4.00) | | 5.00 (0.00, 13.00) | | 0.00 (0.00, 0.00) | | 0.00 (0.00, 0.00) | | 0.00 (0.00, 0.00) | | 0.00 (-5.00, 0.00) | | 0.00 (0.00, 0.00) | |
| TCM | 3.00 (0.00, 14.00) | | 0.00 (0.00, 5.00) | | 8.00 (0.00, 22.00) | | 0.00 (0.00, 0.00) | | 0.00 (0.00, 0.00) | | 0.00 (0.00, 0.00) | | -3.00 (-13.00, 0.00) | | 0.00 (0.00, 0.00) | |
| Comorbidities | 0.00 (-10.00, 5.00) | | 0.00 (0.00, 3.00) | | 5.00 (0.00, 16.00) | | 0.00 (0.00, 0.00) | | 0.00 (0.00, 0.00) | | 0.00 (-7.00, 0.00) | | -3.50 (-14.00, 0.00) | | 0.00 (0.00, 0.00) | |
| Personal experience | 3.00 (0.00, 13.00) | | 0.00 (0.00, 3.00) | | 5.00 (0.00, 16.00) | | 0.00 (0.00, 0.00) | | 0.00 (0.00, 0.00) | | 0.00 (0.00, 0.00) | | 0.00 (-8.00, 0.00) | | 0.00 (0.00, 0.00) | |
| Healthcare service | 12.00 (2.00, 38.00) | | 3.00 (0.00, 12.00) | | 12.00 (3.00, 39.00) | | 0.00 (0.00, 0.00) | | 0.00 (0.00, 0.00) | | 0.00 (0.00, 0.00) | | 0.00 (-8.00, 0.00) | | 0.00 (0.00, 0.00) | |
| Health communication | 1.00 (0.00, 8.00) | | 0.00 (0.00, 0.00) | | 3.00 (0.00, 10.00) | | 0.00 (0.00, 0.00) | | 0.00 (0.00, 0.00) | | 0.00 (0.00, 0.00) | | 0.00 (-3.00, 0.00) | | 0.00 (0.00, 0.00) | |
| Academic research | 3.00 (-2.00, 12.00) | | 0.00 (0.00, 3.00) | | 6.00 (0.00, 15.00) | | 0.00 (0.00, 0.00) | | 0.00 (0.00, 0.00) | | 0.00 (0.00, 0.00) | | 0.00 (-6.00, 0.00) | | 0.00 (0.00, 0.00) | |

Notes: Values are Mean (SD) and Median (Q1, Q3) at the post level.
